# Discrimination of Receptor-Mediated Endocytosis by Surface-Enhanced Raman Scattering

**DOI:** 10.1021/acs.langmuir.1c03305

**Published:** 2022-05-13

**Authors:** Deniz Yılmaz, Mustafa Culha

**Affiliations:** † Faculty of Engineering, Department of Genetics and Bioengineering, Yeditepe University, 34755 Istanbul, Turkey; ‡ Sabanci University Nanotechnology Research and Application Center (SUNUM), Tuzla, 34956 Istanbul, Turkey; § Department of Ophthalmology and Internal Medicine, Morsani College of Medicine, The University of South Florida, Tampa, Florida 33612, United States

## Abstract

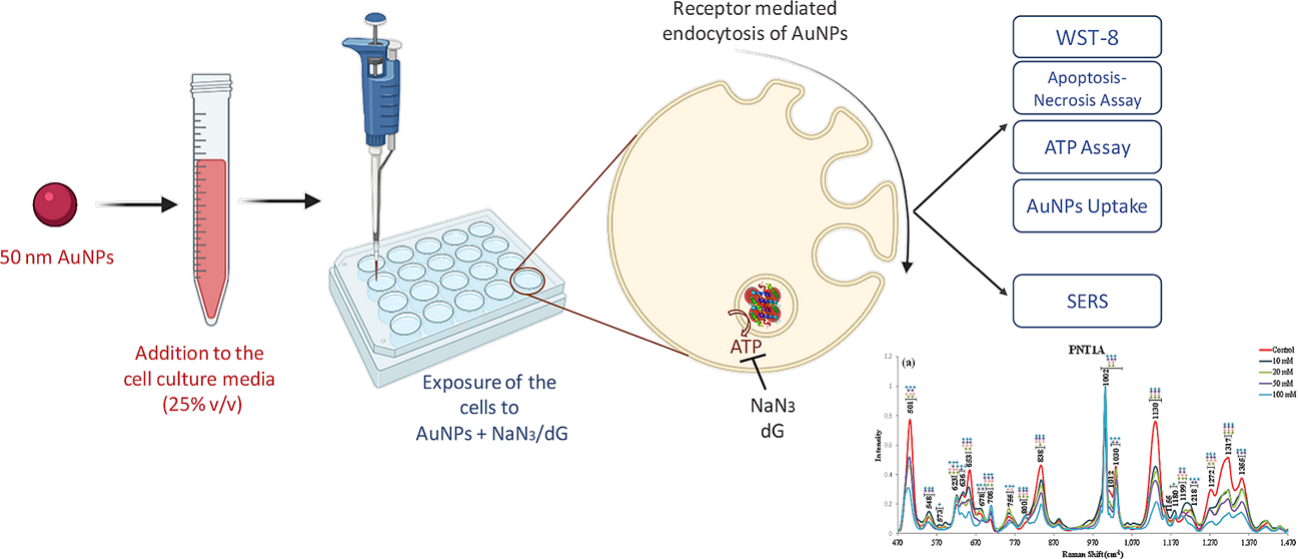

Cellular energy required for the maintenance of cellular life is stored in the form of adenosine triphosphate (ATP). Understanding cellular mechanisms, including ATP-dependent metabolisms, is crucial for disease diagnosis and treatment, including drug development and investigation of new therapeutic systems. As an ATP-dependent metabolism, endocytosis plays a key role not only in the internalization of molecules but also in processes including cell growth, differentiation, and signaling. To understand cellular mechanisms including endocytosis, many techniques ranging from molecular approaches to spectroscopy are used. Surface-enhanced Raman scattering (SERS) is shown to provide valuable label-free molecular information from living cells. In this study, receptor-mediated endocytosis was investigated with SERS by inhibiting endocytosis with ATP depletion agents: sodium azide (NaN_3_) and 2-deoxy-d-glucose (dG). Human lung bronchial epithelium (Beas-2b) cells, normal prostate epithelium (PNT1A) cells, and cervical cancer epithelium (HeLa) cells were used as models. First, the effect of NaN_3_ and dG on the cells were examined through cytotoxicity, apoptosis–necrosis, ATP assay, and uptake inhibition analysis. An attempt to relate the spectral changes in the cellular spectra to the studied cellular events, receptor-mediated endocytosis inhibition, was made. It was found that the effect of two different ATP depletion agents can be discriminated by SERS, and hence receptor-mediated endocytosis can be tracked from single living cells with the technique without using a label and with limited sample preparation.

## 1 Introduction

Adenosine triphosphate (ATP) is the cellular currency to maintain cellular life. A better understanding of ATP-dependent metabolisms is critical for many reasons, including disease diagnosis and treatment.^1^ Endocytosis is an energy-dependent cellular process that is used not only for the internalization of molecules but also for cell signaling, motility, mitosis, growth, and differentiation.^2−4^ Among the endocytosis types, receptor-mediated endocytosis is the most well-known pathway involving receptor–ligand complex formation, membrane invagination, and coated pit and coated vesicle formation steps. After these steps, the obtained vesicle matures into early endosomes and then the late endosomes. Due to its energy-dependent nature, it can be inhibited by ATP depletion agents such as NaN_3_ and dG.^5^ NaN_3_ causes ATP depletion by inhibiting oxidative phosphorylation, the activity of cytochrome oxidase, a mitochondrial transport chain enzyme, and the activity of ATP hydrolase by affecting ATPases such as ABC transporters, preprotein translocase SecA, DNA topoisomerase IIα, and ecto-ATPases.^6,7^ On the other hand, dG depletes ATP by inhibiting glycolysis as it is a glucose analogue. It internalizes into cells like glucose and interferes with glucose metabolism by inhibiting hexokinases and phosphoglucose isomerases.^8,9^ As a result, glycolysis and oxidative phosphorylation are disrupted, and ATP production levels are decreased.^10^ While these inhibitors deplete ATP, they also block receptor-mediated endocytosis. Thus, they can be used as a receptor-mediated endocytosis inhibitor.

Understanding the interaction routes of nanomaterials in biological systems is crucial to investigate the plausibility of the nanomaterials. Thus, SERS has recently started to attract attention as an alternative technique with the potential of providing fingerprint information from dynamic pathways of single living cells without a label and long sample preparation processes. It has been investigated for its feasibility for intracellular studies since 1991.^11,12^ The technique was reported to study the intracellular distribution of gold nanoparticles (AuNPs) and their aggregates,^13^ time-dependent endocytosis,^14^ size- and shape-dependent uptake of nanomaterials, pH changes during endocytosis,^15^ endocytosis types,^16^ cellular metabolism changes including death mechanisms,^17,18^ cellular differentiation,^19^ mitosis,^20^ and DNA damage.^21^ All of these studies suggest that the technique has potential for further investigations of intracellular processes.

In this study, we report the application of the technique to investigate the receptor-mediated endocytosis of AuNPs. For this purpose, an average 50 nm size of spherical AuNPs as SERS substrates and three model cell lines were used: human lung bronchial epithelium (Beas-2b) cells, normal prostate epithelium (PNT1A) cells, and cervical cancer epithelium (HeLa) cells. The receptor-mediated endocytosis was inhibited by NaN_3_ and dG. The inhibition rate of internalization of AuNPs was measured by flow cytometry and ATP depletion was measured with ATP assays. Finally, endocytosis inhibition by ATP depletion was investigated using SERS. The obtained SERS data were analyzed by principal component analysis (PCA) and linear discrimination analysis (LDA).

## 2 Experimental Section

### 2.1 Synthesis and Characterization of Gold Nanoparticles (AuNPs)

AuNPs were synthesized by a modification of the Turkevich method based on citrate reduction.^22^ Ten milligrams of gold(III) chloride trihydrate (HAuCl_4_·3H_2_O) (Sigma Aldrich, Germany) was dissolved in 100 mL of ddH_2_O, and this solution was boiled. When boiling, 1 mL of 1% sodium citrate (Na_3_C_6_H_5_O_7_) (Merck, Germany) was added at once. The obtained solution was boiled for 15 min and kept at room temperature for cooling. The synthesized AuNPs were characterized by dynamic light scattering (DLS) (Zetasizer Nano, Malvern, U.K.) and using a UV/vis spectrometer (Lambda 25, PerkinElmer).

### 2.2 Cell Culture

Normal prostate epithelium (PNT1A), human lung bronchial epithelium (Beas-2b), and cervical cancer epithelium (HeLa) cell lines were purchased from American Type Culture Collection (ATCC). The Beas-2b cell line was cultured in high-glucose Dulbecco’s modified Eagle’s medium (DMEM) (Gibco) supplemented with 5% fetal bovine serum (FBS) and 1% penicillin-streptomycin (PS). PNT1A and HeLa cell lines were cultured in DMEM supplemented with 10% FBS and 1% PS. The cells were cultured at 37 °C under a 5% CO_2_ humidified atmosphere.

### 2.3 Cell Proliferation Assay

For the investigation of cytotoxicity of the inhibitors NaN_3_ and dG, cell proliferation was measured by the WST-8 assay (Abcam). PNT1A, Beas-2b, and HeLa cells were seeded in each well of a 96-well plate with a cell density of 15 000 cells/well in triplicate. The cells were incubated for 24 h for attachment. Then, the cells were exposed to 10, 20, 50, and 100 mM NaN_3_ or dG for 2 h. After 2 h of treatment with the inhibitors, the inhibitor-containing media were removed and fresh media were added to mimic the flow cytometry and SERS measurements. For the cytotoxicity assessment of AuNPs, the cells were treated with 1.6 × 10^15^ AuNPs, as determined in our previous studies^17,23^ without inhibitor treatment. After a total of 24 h of incubation, the cells were washed with phosphate-buffered saline (PBS) once and incubated with a WST-8-containing medium for 2 h at 37 °C under a 5% CO_2_ humidified atmosphere. After 2 h, the supernatant was transferred to another 96-well plate, and absorbance values were measured at 450 nm with a microplate reader (ELx800 Absorbance Reader, Biotek). As a positive control, 10% dimethyl sulfoxide (DMSO) was used.

### 2.4 Apoptosis/Necrosis Assay

For the investigation of the induced cell death mechanism by ATP depletion agents (NaN_3_ and dG), Annexin V-FITC apoptosis and necrosis detection kit from Calbiochem (Merck Millipore) was employed according to the manufacturer’s instruction. PNT1A, Beas-2b, and HeLa cells were seeded in each well of 24-well plates with a cell density of 60 000 cells/well in triplicate. The cells were incubated for 24 h for attachment at 37 °C under a 5% CO_2_ humidified atmosphere. Then, the cells were exposed to 10–100 mM NaN_3_ or dG for 2 h. After 2 h treatment of inhibitors, the inhibitor-containing media were replaced with fresh media and incubated for 22 h. After 24 h of incubation, the cells were detached and collected into Eppendorf tubes with floating cells in the cell culture medium. The samples were washed with 1× PBS and centrifuged at 1500 rpm for 5 min. The cells were suspended in a 1× binding buffer containing 0.5 μl of Annexin V-FITC reagent and 1 μl of PI reagent per sample and incubated in the dark for 15 min according to the manufacturer’s instruction. The cells were counted as 20 000 events and analyzed on a guava easyCyte 5 (Merck Millipore) benchtop flow cytometer. As a positive control, 10% DMSO was used.

### 2.5 ATP Assay

Intracellular ATP was quantified using an ATP determination kit (Abcam) according to the manufacturer’s protocol after treatment with NaN_3_ or dG. Briefly, PNT1A, Beas-2b, and HeLa cells were seeded in each well of 6-well plates with a cell density of 300 000 cells/well in triplicate. The cells were incubated for 24 h for attachment at 37 °C under a 5% CO_2_ humidified atmosphere. Then, the cells were exposed to 10–100 mM NaN_3_ or dG for 2 h. After 2 h of treatment with inhibitors, the inhibitor-containing media were replaced with fresh media and incubated for 22 h. After a total of 24 h of incubation with inhibitors and then AuNPs, the cells were harvested and washed with 1× cold PBS. Then, the cells were resuspended in ATP assay buffer and centrifuged for 5 min at 4 °C at 13 000*g*. The obtained supernatants were collected and mixed with a 1:1 v/v reaction mix, which includes ATP probe and ATP converter and developer mix in ATP assay buffer. The samples were incubated at room temperature for 30 min in the dark. After incubation, the absorbance of the mixture was determined at 570 nm with a microplate reader (ELx800 Absorbance Reader, Biotek). The amount of ATP in the test samples was calculated using the ATP standard curve.

### 2.6 Flow Cytometry

Endocytosis pathways for AuNP internalization were investigated after 2 h treatment of inhibitors and 22 h treatment of AuNPs. Briefly, 60 000 cells/well were seeded in each well of 24-well plates in triplicate and incubated for 24 h for attachment. After 24 h, the cells were exposed to the inhibitors with mentioned concentrations for 2 h, and then inhibitor-containing media were removed for exposure with AuNPs. After removal of inhibitor-containing media, media including 25% v/v AuNPs were added for the inhibition of uptake. For the normalization step, the cells were treated with inhibitors only for 2 h, and then inhibitor-containing media were removed and fresh media were added to the cells. After 24 h of treatment, the media were removed, and the cells were washed and collected. The side scatter shift (SSC) of the cells was analyzed without further staining as 20 000 events on guava easyCyte 5 (Merck Millipore). The SSC shifts of cells with and without AuNPs were measured, and the cells that were not treated with AuNPs were used for the normalization to avoid obtaining SSC shifts only from inhibitors. The control cells were treated with only AuNPs for 22 h, and normalized SSC shift values for cells treated with the inhibitor and AuNPs were compared to the control groups.

### 2.7 SERS Measurements

**Figure 1:**
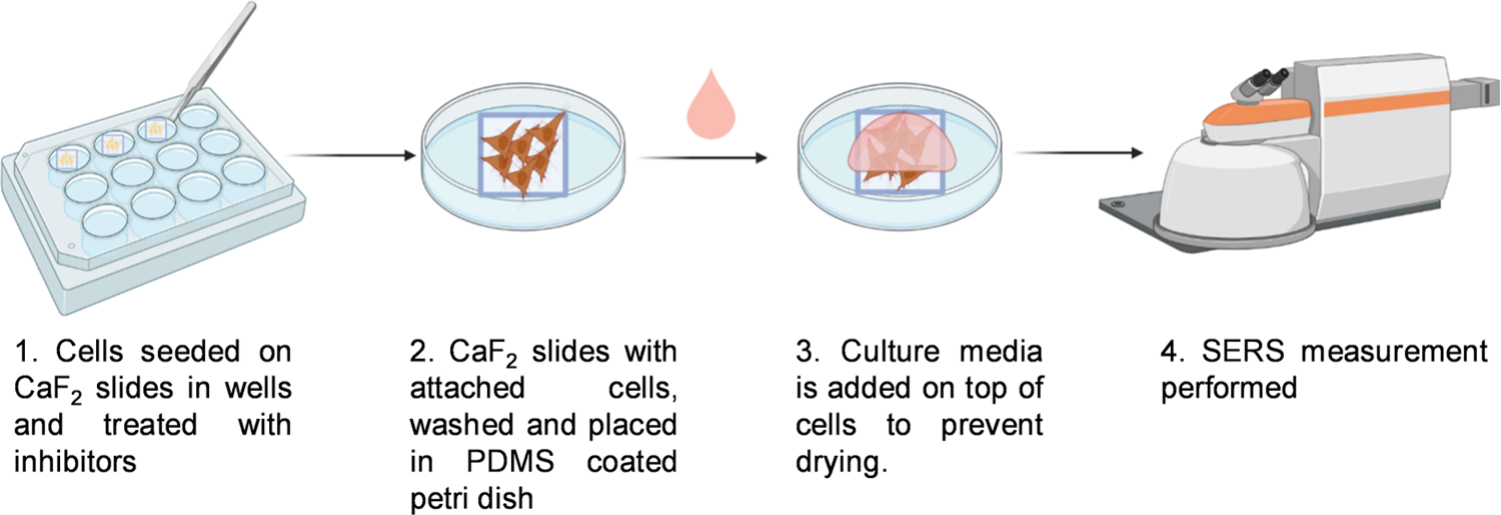
Schematic illustration of SERS measurements from living cells.

PNT1A, HeLa, and Beas-2b cells were seeded in approximately 1 cm^2^ calcium fluoride (CaF_2_) slides in 24-well plates with a cell density of 15 000 cells/well. The cells were incubated and exposed to the inhibitors and AuNPs as mentioned above. Then, AuNP-containing media were removed, and the cells were washed with PBS. CaF_2_ slides, where living cells were attached, were placed on a poly(dimethylsiloxane) (PDMS)-coated Petri dish, which was used to keep CaF_2_ slides with a size of 1 cm^2^ in place and prevent interference from the Petri dish. To keep the cells alive during measurement, 20 μL of cell culture medium was added on top of the cells. The schematic illustration in Figure 1 shows the steps of SERS experiments. For the SERS measurements, a Renishaw inVia Reflex Raman spectrometer equipped with a high-speed encoded stage (Streamline) and a Leica DM2700 Dark Field upright microscope with a 1200 line/mm grating was used. An approximate area of 10 μm × 10 μm on each cell was mapped with a 2 μm step size due to the 2.5 μm laser spot size for the Leica 20× objective with 0.40 NA. The spectra from the cells were collected with a 150 mW laser power and 2 s exposure time in the 470–1470 cm^–1^ spectral range. From each cell, 42–64 spectra were collected depending on the size, position, and spread on the CaF_2_ surface and averaged. In each treatment group, the spectra from a minimum of 10 cells were collected for one experiment, and each experiment for treatment groups was run in triplicate. The obtained spectra from 30 cells for each treatment were averaged and processed with background correction, removal of cosmic spikes, smoothing, and normalization using Wire 4.2 software, as shown in Figure S1.^24^ To investigate the possible spectral interference of inhibitors with the intracellular SERS spectra, the spectra of NaN_3_ and dG at a final concentration of 100 mM were obtained by mixing the inhibitor solutions in cell culture media with 25% v/v colloidal AuNP suspension. A small volume of this mixture was spotted on a CaF_2_ slide and allowed to dry at room temperature before SERS acquisition. A 10 s exposure time and a 150 mW laser power were used to collect 10 spectra from each spot placed on CaF_2_. The spectra from inhibitors were analyzed in the same way as the spectra obtained from cells. The spectral interpretations of the peaks were made cautiously in interference regions (Figure S7). The tentative peak assignments are provided in Table S1.

### 2.8 Statistical Analysis

For the emphasis on the variation of SERS spectra, principal component analysis (PCA) was applied to the obtained average SERS spectra from 30 cells for each treatment group. After the PCA analysis, linear discriminant analysis (LDA) was applied to the obtained PC scores to observe the discrimination of the obtained spectra with treatments of inhibitors. The leave-one-out cross-validation methodology was applied to demonstrate the accuracy of the classification, and the sensitivity and specificity values were calculated from the obtained confusion matrix. Two-tailed Student’s *t*-test was applied to the intensity of desired Raman shift. The samples with a *p* ≤ 0.05 significance value were marked in the results. P values for the selected Raman shift in the SERS spectrum were shown on SERS spectra.

## 3 Results and Discussion

### 3.1 Characterization of AuNPs

**Figure 2:**
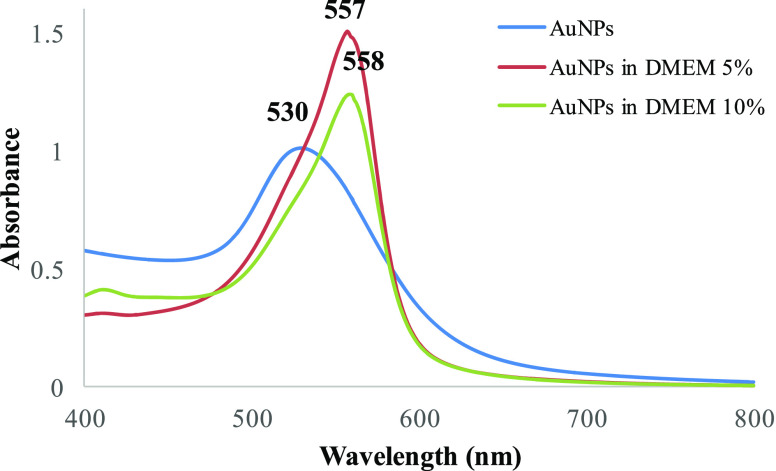
(a) UV/vis spectra of bare AuNPs and in DMEM including 5 or 10% FBS.

The UV/vis and DLS spectra of the synthesized AuNP colloidal suspension are shown in Figure 2 and Table 1. The maximum absorbance of AuNP suspension at 530 nm is observed. Their average hydrodynamic size and ζ potential are found to be 52 nm and −26.8 mV, respectively. As AuNPs are added to the cell culture media, a protein corona is formed on the AuNP surfaces, causing an increase in their size. As they are added to the cell culture media including 5 or 10% FBS, the size increases to 71 and 72 nm, respectively, as indicated by the surface plasmon absorption peak shifts to 557 and 558 nm, respectively. The ζ potential was −24.0 in 5% FBS and −18.5 mV in 10% FBS as a result of the formation of the protein corona, indicating a decrease from −26.8 mV to more positive values.

**Table 1:** Properties of bare AuNPs and in DMEM including 5 or 10% FBS

	λ_max_	hydrodynamic size (nm)	ζ potential (mV)
AuNPs	530	52.26 ± 2.13	–26.8 ± 0.23
DMEM 5% FBS	557	71.16 ± 5.40	–24.0 ± 0.96
DMEM 10% FBS	559	72.28 ± 5.74	–18.5 ± 2.11

### 3.2 Cytotoxicity Assessment

**Figure 3:**
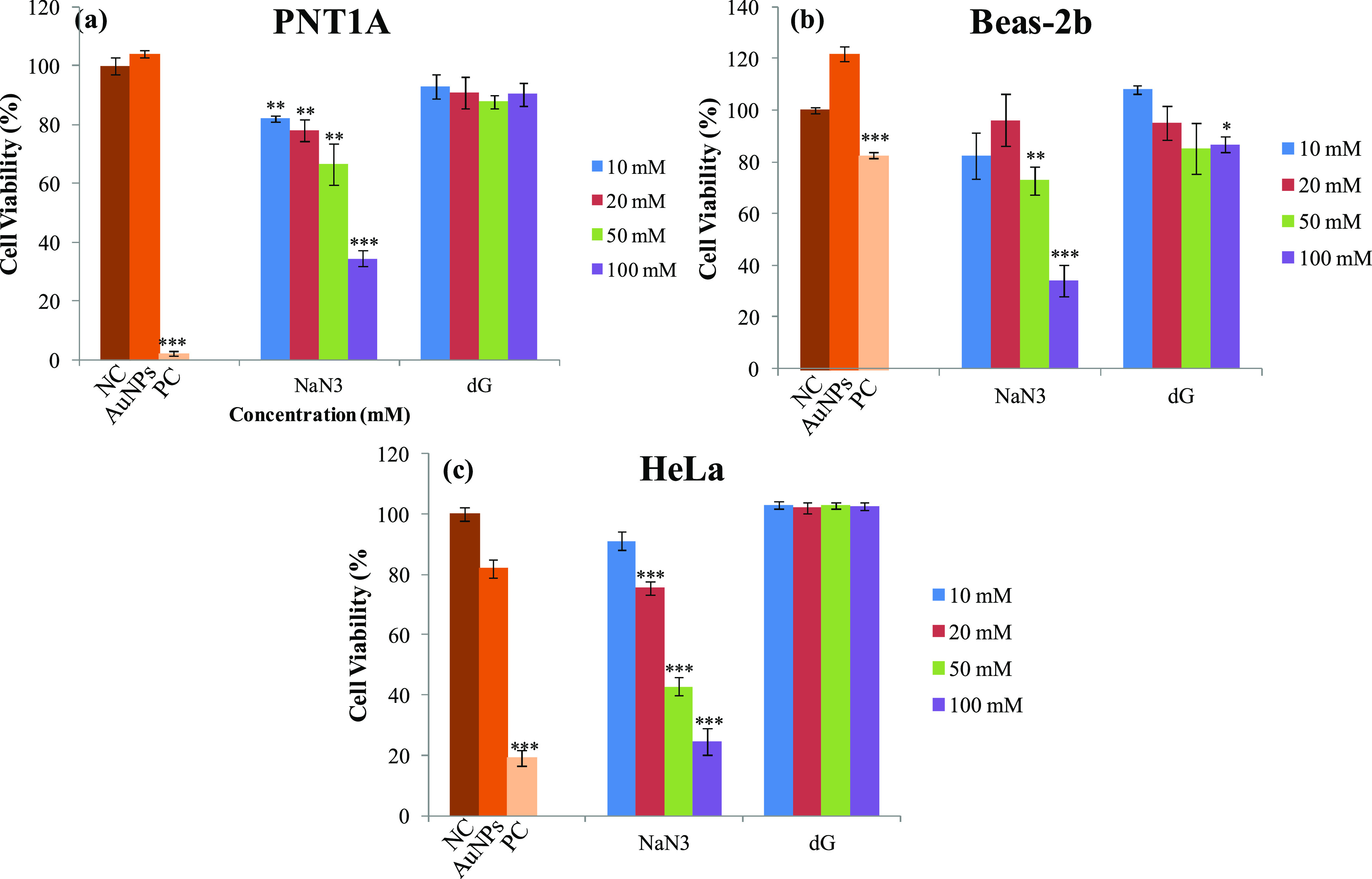
WST-8 cell proliferation results of (a) PNT1A, (b) Beas-2b, and (c) HeLa after 2 h of exposure to 10, 20, 50, and 100 mM NaN_3_ and dG. Statistically significant changes were calculated by two-paired Student’s t-test and marked with asterisks, * for *p* ≤ 0.05, ** for *p* ≤ 0.01, and *** for *p* ≤ 0.001. PC: positive control (10% DMSO was used as a positive control), NC: negative control.

The cytotoxicity of the receptor-mediated endocytosis inhibitors NaN_3_ and dG was assessed with the WST-8 cell proliferation assay, and the obtained data are shown in Figure 3. For the inhibition of endocytosis, it would be better if the inhibitors did not cause any cytotoxicity. Induction of the cytotoxicity will alter the cellular mechanisms rather than endocytosis, and altered dynamics inside the cell could affect the remaining results, especially the obtained intracellular SERS spectra. However, when ATP depletion agents are used as receptor-mediated endocytosis inhibitors, it is not easy to avoid the toxicity effect of all types of inhibitors. Moreover, it should also be noted that using only one type of inhibitor is not recommended because each of the inhibitors has a different mechanism of action and also a different side effect. Thus, here we used two agents, NaN_3_ and dG, and found that NaN_3_ caused a cytotoxic effect on all cell lines with a decrease in the cell viability to 34% for Beas-2b, 24% for HeLa, and 34% for PNT1A, while dG caused the cell viability to decrease only in the Beas-2b cell line to 85%. NaN_3_-dependent toxicity results were used for the evaluation of the intracellular SERS spectra, especially when the spectra differ from the dG-treated spectra. Moreover, cytotoxicity-induced cell death mechanisms were also examined and used to understand their effect on the intracellular SERS spectra.

### 3.3 Apoptosis/Necrosis Assay

In addition to the cell proliferation assay, the apoptosis/necrosis assay was used for the investigation of the effect of the ATP depletion agents on cell death mechanisms due to their cytotoxicity. Figure S2 shows apoptosis/necrosis assay results with the whole cell population (Figure S2c,e,g) and subtracted cell death mechanism induction results (Figure S2b,d,f), which are obtained when the live cell population percent was removed from the results to clearly visualize the apoptosis and/or necrosis induction. The results show that similar to cell proliferation assay results, NaN_3_ causes apoptosis and/or necrosis in cell populations, while dG does not cause a significant effect on cell death mechanisms compared to the control groups. For the Beas-2b cell line, NaN_3_ causes significant necrosis of up to 3.6% of the population from 0.2%, and for the HeLa cell line, the early and late apoptosis are induced up to 5.3% from 0.8 and 4.2% from 0.7%, respectively, with NaN_3_ treatment. For the PNT1A cell line, late apoptosis is induced up to 3.4% in the population from 0.7% with NaN_3_ exposure.

### 3.4 Flow Cytometry

Receptor-mediated endocytosis is inhibited with the use of NaN_3_ and dG, and the inhibition rates are shown as SSC shift decrease from flow cytometry data in Figure S3. As seen, both inhibitors caused a decrease in the uptake of AuNPs in all types of cell lines with differentially decreasing rates via the ATP depletion effect. With the NaN_3_ treatment, AuNP internalization was reduced to 64% for Beas-2b cells, 72% for HeLa cells, and 57% for PNT1A cells. The highest decrease was achieved for the PNT1A cell line with the NaN_3_ treatment. Moreover, the highest decrease in AuNP internalization was achieved with the dG treatment for HeLa cells, and it decreased internalization to 24%. dG also resulted in a decrease in AuNP internalization in PNT1A cells and Beas-2b cells to 58 and 71%, respectively. When inhibitors were used as ATP depletion agents, they caused different uptake decreases due to their mechanisms of action. NaN_3_ depletes ATP by inhibiting oxidative phosphorylation.^6^ It can also affect the *K*_ATP_ channels and cellular morphology and induce apoptosis.^25^ Moreover, dG depletes ATP by inhibiting glycolysis as it is a glucose analogue.^8,9,26^ It could also interfere with cellular metabolisms such as N-linked glycosylation, mitochondrial reactive oxygen species (mROS) production, autophagy, and ER stress.^27,28^ Thus, they can cause varying responses by interfering with different cellular processes.

### 3.5 ATP Assay

**Figure 4:**
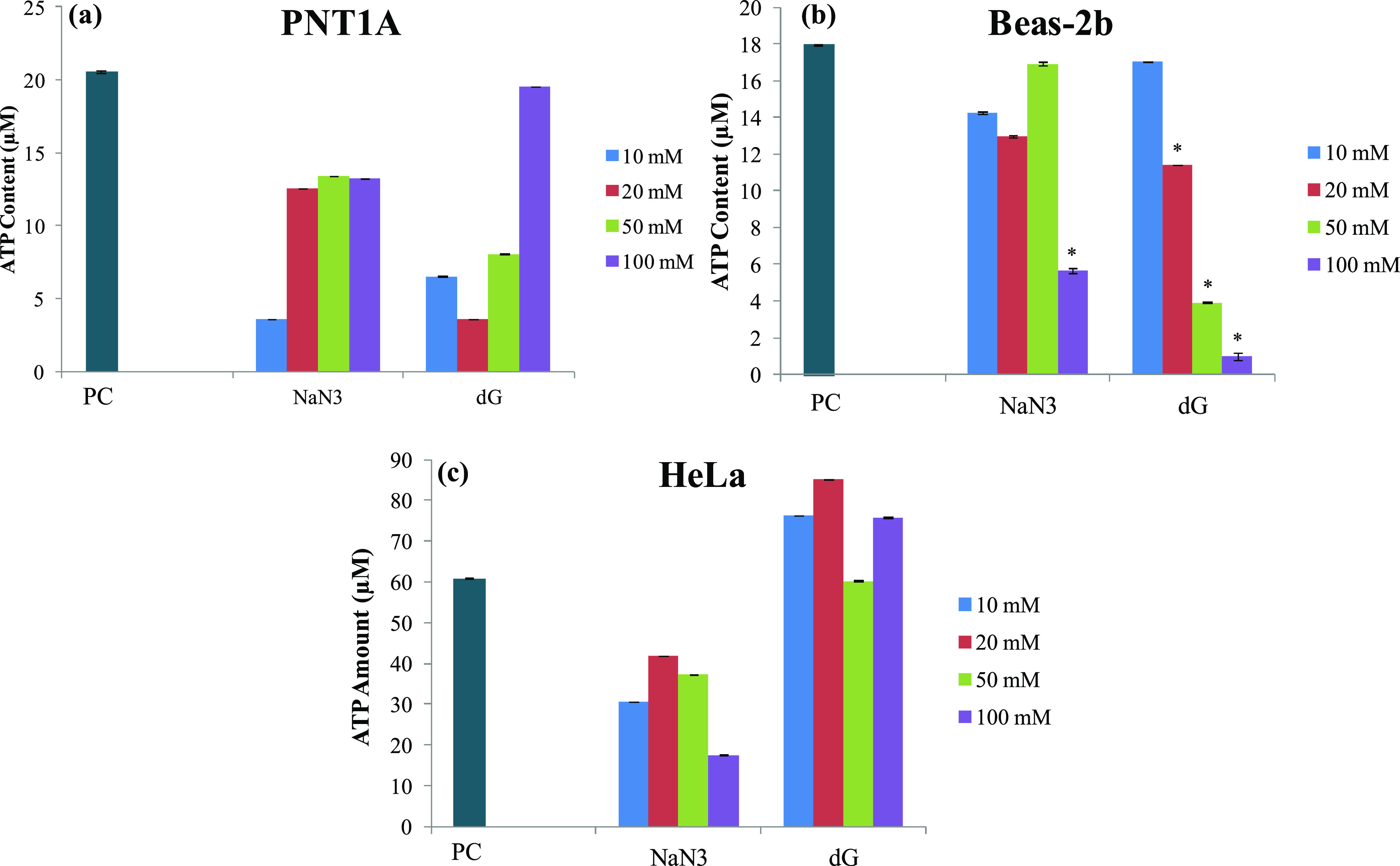
ATP assay results of (a) PNT1A, (b) Beas-2b, and (c) HeLa after 2 h of exposure to 10, 20, 50, and 100 mM NaN_3_ and dG then 22 h of exposure to AuNPs. NC: Negative control. Statistically significant changes were calculated by two-paired Student’s *t-*test and marked with asterisks, * for *p* ≤ 0.05, ** for *p* ≤ 0.01, and *** for *p* ≤ 0.001.

To monitor ATP depletion, a decrease in the ATP concentration in the presence of inhibitors was investigated by ATP assay, and the results are given in Figure 4. It was found that both NaN_3_ and dG caused a significant reduction in the ATP concentration in Beas-2b cells to 5 and 0.9 μM for NaN_3_ and dG, respectively, from a total of 17 μM ATP. For the HeLa cell line, NaN_3_ caused the ATP concentration to decrease to 17 μM from 60 μM, and no ATP decrease was found with dG exposure. For the PNT1A cell line, NaN_3_ decreased the ATP concentration to 13 μM from 20 μM, and dG caused ATP concentration to decrease to 3 μM with 20 mM treatment; no ATP reduction was observed with the highest dose. Being ATP depletion agents, both of the inhibitors were expected to decrease the ATP concentration; however, a significant decrease could not be achieved for PNT1A and HeLa cell lines. On the other hand, when SERS spectra were evaluated with the dose-dependent treatment of the inhibitors, dG provided significant changes for all of the cell lines used. This inconsistency could be explained by the detection limits, interference of nanoparticles with colorimetric assays, and the nature of SERS measurements. It is known that nanoparticles could interfere with colorimetric assays and lead to inaccurate results.^29^ Moreover, SERS was found to provide more specific information from the surrounding environment of nanoparticles when compared with the colorimetric assays.^17^ With the enhancement of Raman signals from molecules in close vicinity of nanoparticles, small changes in the molecular environment of the nanoparticles could be tracked. Even a small change in the microenvironment can affect the overall spectra. SERS may not require overall dramatic changes in external conditions. Furthermore, it should also be noted that most of the assays require many steps, including fixation of cells. However, SERS measurements were obtained from single living cells. Thus, it is possible that some changes that were reflected in the SERS spectra can be lost in the processes of assays. Thus, even though a decrease in the ATP concentration could not be achieved for two cell lines with the ATP assay, significant changes in the SERS spectra show that intracellular changes are reflected on the SERS spectra but not in the ATP assay results.

### 3.6 SERS Measurements

**Figure 5:**
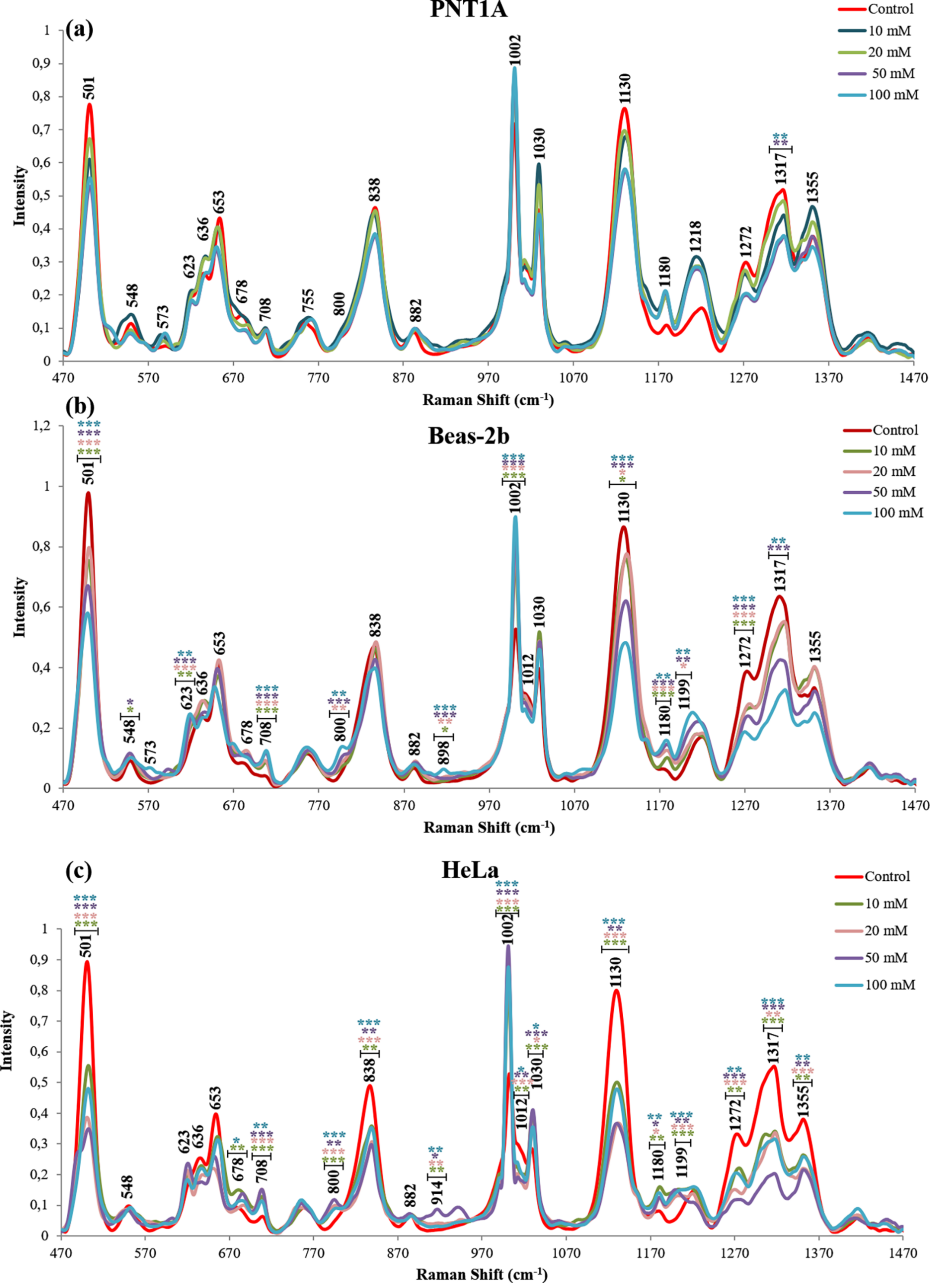
Average SERS spectra of (a) PNT1A, (b) Beas-2b, and (c) HeLa cells exposed to 10, 20, 50, and 100 mM NaN_3_. Statistically significant changes were calculated by two-paired Student’s t-test and marked with asterisks, * for *p* ≤ 0.05, ** for *p* ≤ 0.01, and *** for *p* ≤ 0.001 with the comparison of 10 mM NaN_3_ (green), 20 mM (pink), 50 mM (purple), 100 mM (blue), and the control group.

**Figure 6:**
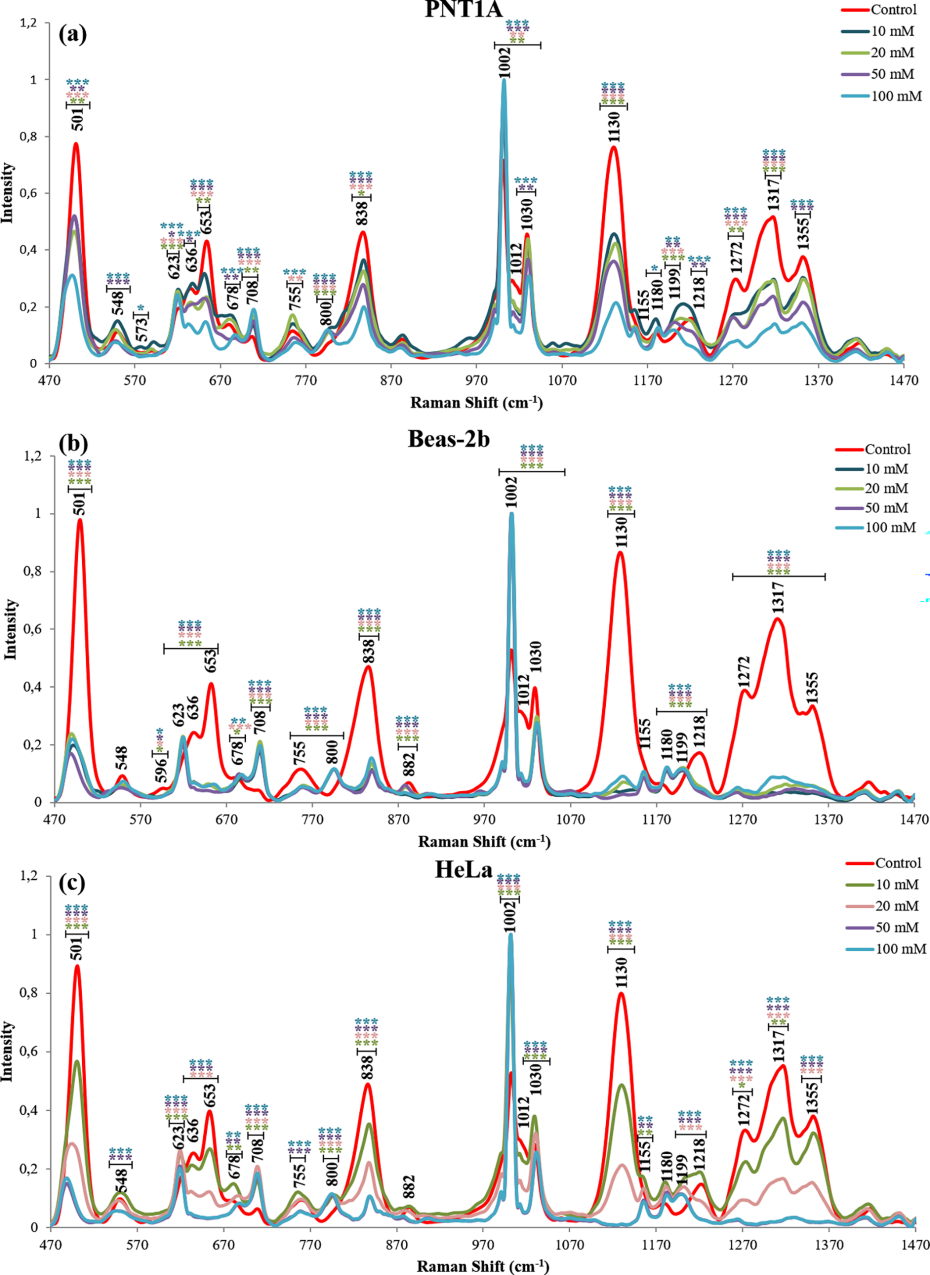
Average SERS spectra of (a) PNT1A, (b) Beas-2b, and (c) HeLa cells treated with 10, 20, 50, and 100 mM dG. Statistically significant changes were calculated by two-paired Student’s t-test and marked with asterisks, * for *p* ≤ 0.05, ** for *p* ≤ 0.01, and *** for *p* ≤ 0.001 with the comparison of 10 mM dG (green), 20 mM (pink), 50 mM (purple), 100 mM (blue), and the control group.

The intracellular SERS spectra from three model cell lines were obtained after their exposure to NaN_3_ and dG, shown in Figures 5 and 6, respectively. However, before explaining the significant alterations of the spectra with inhibitor treatment, the nature of intracellular SERS spectra should be explained.

A comparison of SERS and spontaneous Raman spectra from a living cell is provided in Figure S4. As seen, it is clear that there is no contribution from spontaneous Raman scattering. The SERS scattering overwhelmingly suppresses Raman scattering. The mapping images in Figure S5 suggest that the AuNPs and their aggregates are nonhomogeneously distributed as expected. As AuNPs enter the cell through endocytosis, they accumulate in endosomes. Then, the endosomes mature and fuse with lysosomes for the degradation process. During the maturation, endosomes travel through the cytoplasm to interact with other organelles; thus, AuNPs can be in any location in a living cell.^30^ Note that since SERS spectra mainly originate from the aggregates, spontaneous Raman scattering from the cellular background does not contribute to the overall spectra due to its highly inefficient nature as mentioned above. Furthermore, when we evaluate the variation of the intracellular spectra (Figure S6), which is obtained from approximately 1500 spectra (50 spectra from each cell and a total of 30 cells), it was found that the variations change for each of the treatment group. This is an expected situation because the measurements were obtained from single living cells, which means the effect of the inhibitor treatment could vary from cell to cell. However, when the statistical analysis of the spectra is taken into account as mentioned below, even if there is a variation in the measurements, average spectra can reflect the population, and populations can be discriminated significantly with the inhibitor treatment.

Although some AuNPs can escape from endosomes, the observed spectra are considered mainly from aggregates of AuNPs in endosomes. Kneipp et al. studied the internalization of AuNPs in cells and found that starting from 120 min of exposure time, they form small aggregates as dimers and trimers. As the exposure time increases, they are grown into aggregates.^13,31^ It is worth mentioning here that the presence of large aggregates in the cell explains why a laser line in the NIR region can give an excellent SERS enhancement. Kneipp et al. further demonstrated that the obtained intracellular SERS spectra originate from endosomal structures including proteins, the secondary structure of proteins, amino acids, lipids, and nucleotides due to the accumulation of AuNPs in the endosomes during endosomal pathway development. Their study concluded that the observed spectral changes indicate molecular composition changes in the endosomes as the process is a dynamic one.^13^ In another study, Büchner et al. investigated the cellular uptake of AuNPs and showed that the obtained spectra result from the molecular changes taking place during the endosomal maturation.^32^ They stated that the spectra mostly originated from the protein side chains with varying degrees of interaction with AuNPs during endocytosis. Ando et al. used the scattered light from the AuNP aggregates localized in cells to acquire SERS spectra in a time- and motion-dependent manner and showed that spectral changes resulted from the motion of nanoparticles occurring by transportation, accumulation, and digestion during endocytosis.^30^ In another example, Fujita et al. stated that the observed intracellular SERS spectra originated from molecular changes in the microenvironment of AuNPs as a result of their motion during endocytosis.^33^ In the light of these previous reports, the changes in the intracellular spectra can be related to the endosomal structural changes with the inhibition of receptor-mediated endocytosis. In the inhibition process, the development of the pathway is blocked, which results in the accumulation of some proteins or blockage of the recruitment of new proteins and molecular species. These changes occur on the endosomal membrane, where AuNPs are found very close, a few nanometers away. Thus, the spectral changes could be interpreted based on the molecular changes during endosomal pathways.^13,30,32,33^

When inhibition with NaN_3_ is considered, it is known that NaN_3_ causes ATP depletion by the inhibition of oxidative phosphorylation as a result of the inhibition of cytochrome oxidase, a mitochondrial transport chain enzyme.^6^ Moreover, NaN_3_ affects *K*_ATP_ channels and cellular morphology and can induce apoptosis.^25,34^ On the other hand, dG depletes ATP by inhibiting glycolysis as it is a glucose analogue. It internalizes into the cells similar to glucose and interferes with glucose metabolism by inhibiting hexokinases and phosphoglucose isomerase.^8,9^ As a result, glycolysis and oxidative phosphorylation are disrupted and ATP production is decreased.^10^ It can also interfere with N-linked glycosylation and induce mitochondrial reactive oxygen species (ROS) formation, autophagy, and ER stress.^27,28^

In addition, endocytosis requires energy for the movement, acidification, and fusion of vesicles and coordination of these events to achieve the internalization of particles.^35^ Thus, when AuNPs are internalized by the cells in the presence of NaN_3_ or dG, the biochemical surroundings of AuNPs are changed, and these changes are reflected in the SERS spectra.

When NaN_3_ and dG are used for ATP depletion, endocytosis is affected in different steps. In the early steps of endocytosis, ATP is used for the coated pit assembly and vesicle budding.^35^ When the coated pit assembly and vesicle budding is blocked, the vesicle cannot be scissored from the membrane and coated vesicles cannot be formed. Thereby, the maturation into the late endosomes and merging with the lysosomes to form endolysosomes is inhibited. When this dynamic structure is interrupted with an inhibitor, the dynamic surrounding environment of the internalized AuNPs is expected to change, and these changes are reflected on the obtained SERS spectra. The inspection of the SERS spectra reveals that the intensity of the peaks at 501 cm^–1^ (S–S) and 1130 cm^–1^ (phospholipid) significantly decreases in all SERS spectra regardless of the cell type used in the study. In the literature, it was shown that the intensity change of the peak at 501 cm^–1^ could be related to the formation or deformation of endosomes based on cysteine-rich proteins^14^ and the intensity decrease at 1130 cm^–1^ originating from phospholipids could be related to the decreased number of the formed endosomes in the presence of endocytosis inhibitors.^16^ Furthermore, in the late steps of the endocytosis, early endosomes mature and late endosomes are formed with the exchange of membrane components, change in the fusion partners, formation of additional intraluminal vesicles (ILVs), drop in the luminal pH, acquisition of lysosomal components, and change in morphology and movement to the perinuclear area.^36,37^ Luminal pH is regulated by the concentration of vacuolar ATPases (V-ATPase) in the membrane, selection of V-ATPase isoforms, and association and dissociation of the subunits of V-ATPases. Subunits of V-ATPase are V0, which serve as a transmembrane pore for protons, and V1, which binds and hydrolyzes the ATP as a soluble part.^38^ V-ATPases are responsible for the accumulation of H^+^ inside the lumen of the endocytic vesicle in an ATP-dependent manner.^39^ Intravesicular acidification obtained by V-ATPases is required for the recruitment of proteins used for the maturation of endocytic vesicles such as Arf1, GTPases, and coat protein complex (COP).^40^ On the other hand, regulatory events for the maturation of early endosomes to late endosomes, such as Rab5/Rab7 conversion and PI conversion, cannot be achieved, which requires the involvement of many proteins, exchange in the membrane components, change in the fusion partners, and a drop in the pH, and this situation can be observed as changes in the intensity of peaks originating from proteins on SERS spectra. Figures 5 and 6 show the average SERS spectra of PNT1A, Beas-2b, and HeLa cells exposed to the NaN_3_ and dG inhibitors at 10, 20, 50, and 100 mM. When the spectra are inspected, it can be seen that the intensities of the peaks at 708 cm^–1^ (Met) and 1002 cm^–1^ (Phe) increase, while those at 1012 cm^–1^ (Trp), 1030 cm^–1^ (Phe), 1271 cm^–1^ (protein, α-helix), 1317 cm^–1^ (amide III, α-helix), and 1355 cm^–1^ (protein, β-sheet) decrease significantly. The changes in these peak intensities on the spectra of the cells treated with both inhibitors indicate that they are independent of cell type and inhibitor mechanisms. Since both NaN_3_ and dG inhibitors cause this effect and they are receptor-mediated endocytosis inhibitors, the observed intensity increase and decrease of these peaks can be due to the changes in the protein profile on the AuNP surfaces during the endosome maturation.

The acidification by V-ATPase provides unique acidic lysosomal pH of ∼4.5, which is essential for the enzyme function. The acidic environment also provides conformational changes for the inactive enzymes.^41^ Furthermore, protease activity in an acid-dependent manner is also crucial for processing the maturation of precursor proteins.^39^ Moreover, ATP depletion can also affect the F-actin assembly and cause unregulated actin polymerization, which are the fundamental processes for the development of endocytosis including membrane invagination, vesicle formation, and movement of endosomes, where many different proteins are used for the initiation and maturation of mentioned steps.^42^ The ATP depletion disrupts V-ATPase activity and actin polymerization, resulting in changes in the protein profile. Such a change in protein profiles in the endosomes strongly affects the SERS spectra during endosomal pathway maturation.^32^ Thus, when the obtained SERS spectra are compared to those of the control groups, the protein peaks at 636 cm^–1^ (protein), 653 cm^–1^ (Tyr), and 1218 cm^–1^ (protein) disappear, and the peaks at 623 cm^–1^ (protein), 1155 cm^–1^ (protein), 1180 cm^–1^ (Tyr, Phe), and 1199 cm^–1^ (Trp) appear as a result of the altered protein profile in the environment of AuNPs and their aggregates in the endosomes. The altered protein profile can be observed at the two spectral regions of 623/636–653 cm^–1^ and 1218/1155, 1180, 1199 cm^–1^, where these peaks are attributed to proteins.

dG and NaN_3_ not only interfere with the endosomal pathway by ATP depletion but also interrupt other pathways including cellular death mechanisms, actin polymerization, mitochondrial activity, and glycolysis.^43^ It should also be noted that the remaining V-ATPases are also localized in the plasma membrane used for the H^+^ balance and secretory vesicles used for physiological homeostasis regulation by the activity of secreted hormones.^39^ These changes are expected to be reflected on SERS spectra when AuNPs are able to escape from the endocytic vesicles and accumulate in the cytosol as individual NPs or aggregates in addition to the endocytosis.^17^

NaN_3_ inhibits mitochondrial ATP production, being an electron transport inhibitor. Moreover, it reduces mitochondrial activity and increases mitochondrial ROS, resulting in mitochondria-mediated apoptosis.^6^ NaN_3_-induced apoptosis can be seen in Figure S2. It also causes morphological changes in cells. In such cells, nuclei shrinkage and increased chromatin condensation are observed.^6^ These alterations in cellular mechanisms are reflected in the SERS spectra. For instance, the intensities of the peaks attributed to proteins at 573 cm^–1^, and peaks at 914 and 814 cm^–1^ attributed to ribose and adenine, respectively, were found to change significantly with only NaN_3_ treatment, which could be due to the activation of apoptosis. NaN_3_ causes the upregulation of Bax and cytochrome c and the downregulation of Bcl-2 and procaspase,^6^ which are all related to apoptosis.^44,45^ With the activation of apoptosis, caspases are activated, leading to the cleavage of several bio-macromolecular structures in the cytoplasm and nucleus including DNA and cytoskeletal proteins. The peaks attributed to cholesterol, lipids, and nucleotides on the intracellular SERS spectra of the cells stimulated with chemotherapy drugs were reported to change significantly due to the endolysosomal membrane destabilization, caspase activation, and apoptosis.^23^ Kuku et al. also showed that the exposure of cells to nanomaterials could cause significant changes in the peaks attributed to amino acids, secondary structures of proteins, nucleotides, and lipids as AuNPs internalized through endocytosis.^17^ Thus, with morphological changes and alterations in the protein profile as well as nuclear structure, it is not surprising to observe spectral changes attributed to proteins and nuclear structures.^46^

In addition to the general changes in the SERS spectra with NaN_3_ treatment, cell type-dependent changes were also observed, as seen in Figure 5. When the SERS spectra of different cell lines were compared, it can be seen that the most affected cell line is HeLa with the highest decrease in the peak intensities and the least-affected cell line is PNT1A with very few significant peak intensity changes. When changes in the peak intensities are compared, peak intensities attributed to ribose (914 cm^–1^) and proteins (1012 cm^–1^ and 1030 cm^–1^) are found to change only for HeLa, and the peak intensities attributed to nucleotides (573 cm^–1^ and 898 cm^–1^) change only for Beas-2b, whereas peak intensities attributed to phosphate ions (800 cm^–1^) and proteins (1199 cm^–1^ and 1355 cm^–1^) change only in the case of PNT1A. These observations suggest that NaN_3_ affects cells in different ways depending on their type. Although NaN_3_ treatment causes cytotoxicity (see Figure 3) and ATP decrease (Figure 4) in all cell lines, it significantly induces apoptosis in both Beas-2b and HeLa cells, while it does not cause a significant increase in the apoptosis rate of PNT1A cells at the highest concentration of NaN_3_, as in the Figure S2. The cellular changes indicate that NaN_3_ causes the activation of different cellular death mechanisms in Beas-2b as necrosis and in HeLa as early and late apoptosis.

With the activation of necrosis, cell integrity is lost and organelles are disintegrated, resulting in the release of cellular content and AuNPs within.^47^ This disintegration can be observed from the spectral changes on the cellular SERS spectra through the appearance of new peaks only observed with necrosis activated with NaN_3_ exposure. It is possible to observe this effect in Beas-2b cells at all NaN_3_ doses. The peak at 573 cm^–1^ attributed to cytosine and guanine and the peak at 898 cm^–1^ attributed to adenine appear only on the spectra of Beas-2b cells exposed to NaN_3_, especially at high doses, when necrosis is significant. The appearance of the peaks on cellular SERS spectra attributed to nucleotides was reported to be the result of DNA fragmentation during cell death.^18^ Thus, the cell type-dependent effect of the NaN_3_ can be explained with the observation of differential changes in the spectral pattern.

When the cells are treated with dG, dG can not only cause the depletion of cellular energy but also inhibits glycolysis, induces mitochondrial ROS formation and autophagy, and interferes with N-linked glycosylation.^27,48^ When compared to NaN_3_ exposure, more dramatic changes were observed on the SERS spectra as seen in Figure 6 for all three cell lines with dG exposure. The peaks attributed to proteins at 755, 838, 882, and 1155 cm^–1^ significantly change only with the dG exposure. Moreover, only the peak intensity at 1155 cm^–1^ is found to increase, while the rest of the peaks decrease. As shown by Büchner et al., structural changes in the proteins during the endosomal maturation can be reflected in the SERS spectra.^32^ Thus, with the interruption of the endocytosis process, obtained changes could be related to the disrupted recruitment and disassembling of required proteins for different metabolisms in the cells in addition to endocytosis, leading to changes in the protein profile in the whole cell with dG exposure.

As an important cellular metabolism, dG can inhibit glycolysis as it is a glucose analogue. This inhibition process starts with the internalization of dG by glucose transporters (GLUTs). Then, dG is phosphorylated by hexokinases (HK) to form 2-deoxy-d-glucose-6-phosphate (dG-6-P). The formed dG-6-P cannot be metabolized via glycolysis and accumulates in the cell. It inhibits HK noncompetitively and phosphoglucose isomerase (PGI) competitively.^8,9,26^ With the inhibition of the first steps of glucose metabolism, glycolysis and oxidative phosphorylation are disrupted, the ATP production level is decreased, and cell growth is inhibited.^48^ On the other hand, the inhibition of glycolysis leads to autophagy with the activation of AMP-activated protein kinases **(**AMPK), which is the result of the decreased levels of ATP and increased levels in the AMP/ATP ratio and ER stress. In ER, dG causes increased Ca^2+^ efflux, which induces elevated cytoplasmic Ca^2+^ concentrations.^49^ This situation activates Ca^2+^/calmodulin-dependent protein kinase β (CaMKKβ) and its downstream target AMPK, which results in autophagy induction.^50^ Another marker of autophagy, Beclin-1, is also activated by dG, which disengages Beclin-1 from Bcl-2 acting as a key antiapoptotic regulatory protein of the mitochondrial death pathway and also negatively regulates Beclin-1.^28^

dG interferes with not only glycolysis but also N-linked glycosylation with its structural similarity to mannose. It is converted to dG-GDP before competing with mannose-GDP during the initial steps of N-linked glycosylation. Conversion of dG to dG-GDP causes a depletion of the chain-forming precursor and causes a further disrupted oligosaccharide formation, which causes the formation of disrupted and unfolded/misfolded glycoproteins, which in turn leads to ER stress at the end.^9^ Formation of unfolded/misfolded glycoproteins in the ER activates unfolded protein response (UPR) and leads to ER stress and death.^51^ UPR has a defensive function to relieve ER stress with the inhibition of protein translation, reduction of the amount of protein entering ER, and to increase in the degradation of aberrant proteins.^52^ However, with increased ER stress, ER stress-specific apoptotic response elements such as C/EBP homologous protein (CHOP) are activated and cause ER stress-induced cell death.^51^

During glycolysis, Ca^2+^ efflux metabolisms are disrupted with dG treatment and ER stress and autophagy are induced. As a result, the whole cellular protein profile changes, and this is reflected as changes in peak intensities originating from proteins (755, 838, 882, and 1155 cm^–1^) as shown in the literature as the significantly altered cellular SERS spectra after the disrupted Ca^2+^ metabolism and induced cell death.^23^

Furthermore, the specific spectral changes are also observed with dG exposure, which are not observed with NaN_3_ exposure such as a decrease in the intensity of the peaks at 548 cm^–1^ (cholesterol) and 596 cm^–1^ (phosphatidylinositol). The change in cholesterol concentration could be related to two different metabolisms: endocytosis and cholesterol efflux. When apoptosis is induced by ER stress with dG exposure, caspase activity and cytosolic Ca^2+^ increase, leading to cholesterol release and phospholipid degradation from the endolysosomal membrane.^53^ This can be observed on SERS spectra as changes in the cholesterol attributed peaks as shown by Altunbek et al.^23^ As a second mechanism, it is known that dG cause conformational changes in the ATP-binding cassette protein A1 (ABCA1), which has role in the cholesterol homeostasis and HDL metabolism.^54^ This change could cause spectral changes at the peak intensities attributed to the cholesterol as well.

The changes in the phosphatidylinositol (596 cm^–1^) and also phosphate ion interactions (800 cm^–1^) could be caused by the altered phosphatidylinositol (PI) metabolism because dG induces Akt phosphorylation independently from altered glucose metabolism, and Akt phosphorylation is required for the phosphatidylinositol-3-kinase activity (PI3K).^55^ PI3K is used for the formation of PI3P, which is used for the regulation of endosomal maturation. PI3P is found on the cytosolic leaflet of EE membranes formed by a PI3K, which is VPS34.^56^ VPS34 forms a complex with p150 and Beclin-1, affected by dG directly,^28^ for the regulation of the VPS34 kinase activity.^57^ With the help of a complex formed, Rab5/Rab7 switch can be achieved, which is among the fundamental process for endosomal maturation.^58^ This alteration in the PI and endosomal maturation metabolism could be observed on the SERS spectra as the decreased intensity of the peaks corresponding to PI (596 cm^–1^) and also an increase in the phosphate ion interactions (800 cm^–1^).

When cell-based differences obtained by dG treatment are taken into account, it can be said that the peak at 573 cm^–1^ originating from cytosine and guanine decreased only for PNT1A, the peak at 596 cm^–1^ originating from phosphatidylinositol decreased only for Beas-2b, and the peak at 882 cm^–1^ did not change only for PNT1A cells. These cell-dependent changes can be caused by the differential effect of dG on each type of cell line. For instance, from the dG treatment, the most affected cell line is Beas-2b and the least-affected cell line is PNT1A. With dG treatment, the uptake rate significantly decreased in all cell lines, and ATP amount decreased significantly in only the Beas-2b cell line. Even though the uptake rate decreased the most in the HeLa cells, Beas-2b cells were more affected by the cytotoxic effect of dG. When cytotoxicity results (Figure 3) are inspected, it can be seen that dG causes cytotoxicity only on Beas-2b cells at the highest concentration, and apoptosis is induced most on the Beas-2b cell line as the induction of both early and late apoptosis when compared to the other cell lines. This means that even though AuNPs internalized by endocytosis and endocytosis are affected by ATP depletion, major cellular metabolism changes such as cell death mechanisms affect cellular SERS spectra compared to any other effects.

### 3.7 Statistical Analysis

**Figure 7:**
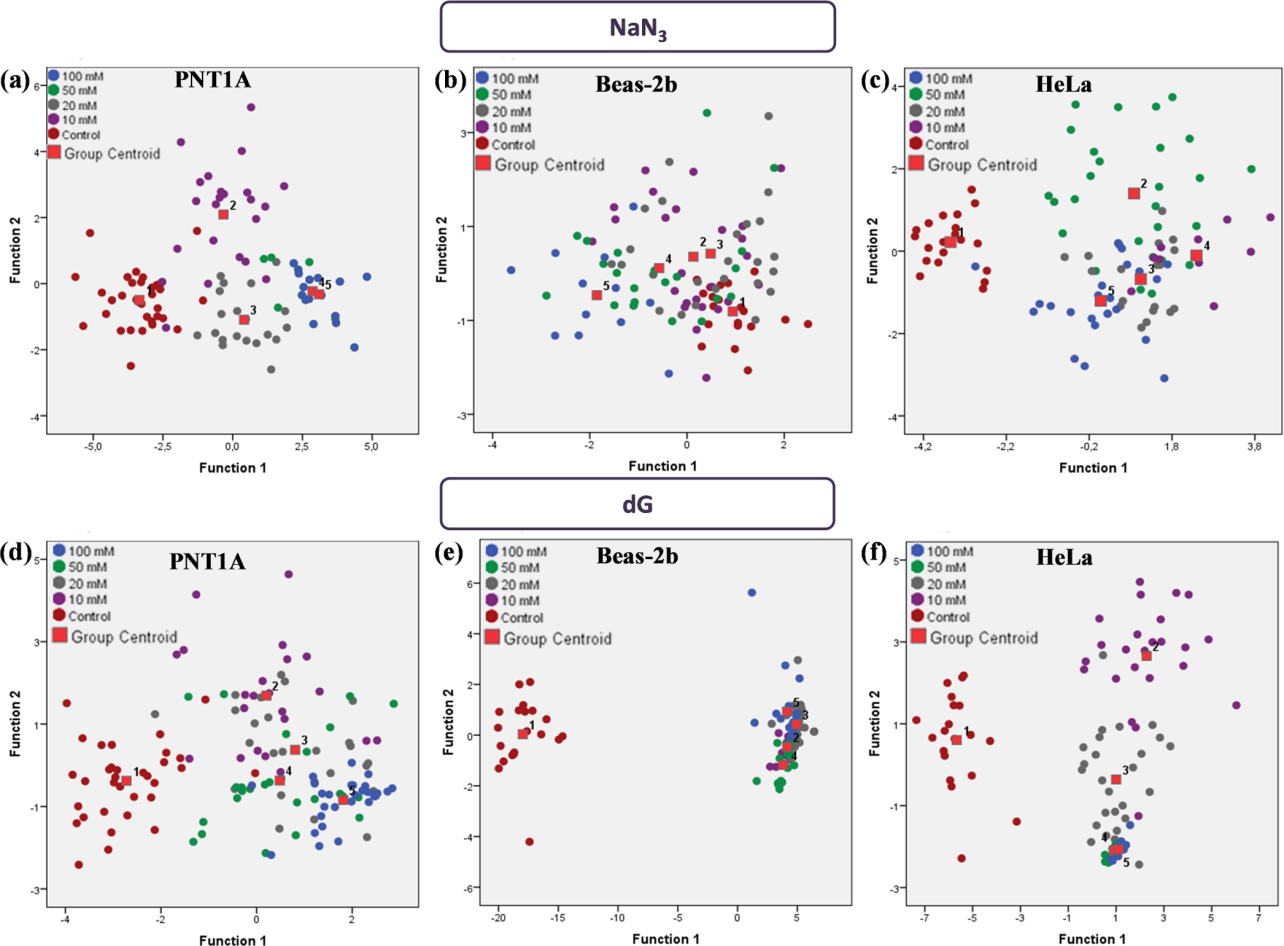
Canonical discriminant function plots of (a, d) PNT1A, (b, e) Beas-2b, and (c, f) HeLa cells exposed to (a–c) NaN_3_ and (d–f) dG. 10–100 mM NaN_3_ and dG were used for treatment and the control group was treated only with AuNPs.

To demonstrate the variation in SERS spectra as cells are exposed to NaN_3_ and dG, PCA and LDA were applied to the obtained average cellular SERS data. The LDA plots along with the extracted canonical function plots are given in Figures 7 and S8. The LDA provides classified discrimination of selected groups while canonical discriminant analysis (CDA), which is extracted from the LDA analysis results, provides separation of groups by showing the group centroids. As seen from both LDA and CDA plots, the control group is mostly separated from the treatment groups for both of the inhibitors. For the NaN_3_ exposure, the group centroids are achieved separated for HeLa and PNT1A cell lines consistent with the changes in the SERS spectra. The most significant changes are observed on the HeLa cell line with the NaN_3_ exposure, and this is reflected in the LDA and CDA plots. For the Beas-2b cell line, a good separation is not achieved on the LDA plots, but CDA plots showed separation of the groups. For the dG exposure, the Beas-2b treatment provided direct separation of the treatment groups and the group centroids from the control group, but the separations among the treatment groups were weak due to the weak signal obtained from the cells after dG treatment. When there are not enough AuNPs inside the cells, the SERS spectra are not meaningful. The obtained spectra resemble each other without almost any enhancement. This results in the nonseparated treatment groups and the group centroids. When HeLa and PNT1A cells were treated with the dG, HeLa cells provided a better separation. The centroid separation was also observed with PNT1A cells even though the group separation was weak.

Although plots obtained from PCA-LDA can provide significant information from the classification of the treatment groups, they can only provide qualitative information. Thus, we have also calculated the sensitivity, specificity, and accuracy values for each treatment group using the leave-one-out cross-validation method, as shown in Table 2. It was observed that populations of each treatment group for two inhibitors and three cell lines showed good specificity and accuracy, which were between 81.71–100% and 71.84–100%, respectively. However, sensitivity values were found to vary in a broad range. For instance, when PNT1A cells were treated with NaN_3_, the sensitivity value of the 50 mM treatment group decreased up to 38.10%. This situation can be explained in the correlation of averaged SERS spectra. As seen in Figure 5, the 50 mM NaN_3_ treatment group could not be separated from the 100 mM treatment group even in the spectra. Thus, groups in the canonical discriminant plots were overlapped and sensitivity decreased. Moreover, NaN_3_ treatment of the Beas-2b cell line provided the least sensitivity value in the 10 mM treatment group. Similar to PNT1A spectra, SERS spectra obtained from the treatment with 10 and 20 mM NaN3 overlapped, and thus discrimination could not be achieved from the analysis. On the other hand, HeLa cells provided the best classification of the groups with the highest sensitivity values, even though groups were not clearly separated in the spectra. Moreover, when PNT1A cells were treated with dG, the sensitivity decreased up to 44% with 20 mM treatment. As seen in Figure 6, average SERS spectra of 20 mM dG-treated cells overlapped with the 10 mM treated group, and this caused decreased sensitivity. Consequently, when overall inhibitor-treated SERS spectra are compared with control spectra, some of the concentrations overlap in the spectra with the closest concentration and could not provide significant discrimination. However, spectral changes caused by inhibitor treatment can be tracked significantly in a dose-dependent manner, which is directly related to the inhibition of endocytosis and the mechanisms of inhibitors. Moreover, it can also be said that the average spectra reflect the populations and are used for tracking dose-dependent inhibition of endocytosis by SERS.

**Table 2:** Leave-One-Out Classification Results in Terms of Sensitivity, Specificity, and Accuracy

	PNT1A									
	NaN_3_ concentration (mM)					dG concentration (mM)				
	CTRL	10	20	50	100	CTRL	10	20	50	100
sensitivity (%)	97	87	85	38	81	94	64	44	54	90
specificity (%)	98	98	100	95	86	97	94	94	89	91
accuracy (%)	97	96	97	84	85	96	89	84	82	90

	Beas-2b									
	NaN_3_ concentration (mM)					dG concentration (mM)				
	CTRL	10	20	50	100	CTRL	10	20	50	100
sensitivity (%)	85	12	44	33	75	100	59	61	63	53
specificity (%)	80	94	87	82	90	100	83	89	94	93
accuracy (%)	81	74	77	72	88	100	79	82	88	85

	HeLa									
	NaN_3_ concentration (mM)					dG concentration (mM)				
	CTRL	10	20	50	100	CTRL	10	20	50	100
sensitivity (%)	100	70	83	73	83	100	88	65	87	76
specificity (%)	99	99	87	100	93	100	99	97	93	89
accuracy (%)	99	91	86	97	91	100	96	89	92	87

## 4 Conclusions

In this study, receptor-mediated endocytosis of AuNPs from single living healthy and cancerous cells was investigated by SERS. The receptor-mediated endocytosis was inhibited by the ATP depletion agents NaN_3_ and dG. It was found that NaN_3_ caused dose-dependent cytotoxicity on all cell lines, while dG did not cause any cytotoxicity. The toxicity of NaN_3_ induced early and late apoptosis and necrosis in all cell lines. Both inhibitors caused decreased uptake in all cell lines at varying rates. The decrease in ATP concentration was also examined since ATP depletion agents for receptor-mediated endocytosis inhibition were used. When the cellular SERS spectra were compared, the effect of both agents could be considered as related to not only interrupted endosomal pathways but also other cellular metabolisms such as cytoskeleton remodeling, mitochondrial activity, glycolysis, and cellular morphology. The intensities of certain peaks were found to be significantly altered in all SERS spectra regardless of the cell and inhibitor type, which could be the result of the recruitment failure of proteins to the endosomal region required for the maturation of early endosomes to late endosomes and endolysosomes. On the other hand, NaN_3_- and dG-specific spectral changes were also observed with the treatment. These results showed that even with the same inhibition purpose as ATP depletion, metabolic effects of two different agents could be discriminated by SERS and the effects, not only on the endosomal pathway but also on other cellular metabolisms, could be tracked by SERS from single living cells without any labeling and with limited sample preparation.

## Supporting Information Available

Apoptosis/necrosis assay results, data analysis steps, AuNP uptake data, comparison of SERS and Raman spectra of living cells, representative map data of cells treated with inhibitors, SERS spectra of NaN_3_ and dG, LDA plots, and tentative peak assignment table (PDF)
