# Supplementary material for: Discrimination of Receptor-Mediated Endocytosis by Surface-Enhanced Raman Scattering
Source: Langmuir. 2022 May 13;38(20):6281–94. doi: 10.1021/acs.langmuir.1c03305 (PMC9134499; doi:10.1021/acs.langmuir.1c03305)
Supplement: Supplementary file 1 — la1c03305_si_001.pdf [file la1c03305_si_001.pdf]

SUPPORTING INFORMATION

Discrimination of Receptor Mediated Endocytosis by Surface-enhanced Raman Scattering

Deniz Yılmaz[1], Mustafa Culha[2,3*]

[1]Yeditepe University, Faculty of Engineering, Department of Genetics and Bioengineering, 34755, Istanbul, Turkey

[2]Sabanci University Nanotechnology Research and Application Center (SUNUM), 34956, Tuzla, Istanbul, Turkey

[3]Department of Ophthalmology and Internal Medicine, Morsani College of Medicine, The University of South Florida, 33612, Tampa, FL, USA

***Corresponding Author:** Prof. Dr. Mustafa Culha

Telephone: +90 (216) 483 9000

Fax: +90 (216) 483 9885

e-mail: mculha2@gmail.com

Address: Sabanci University Nanotechnology Research and Application Center (SUNUM), Tuzla, Istanbul, Turkey

RESULTS

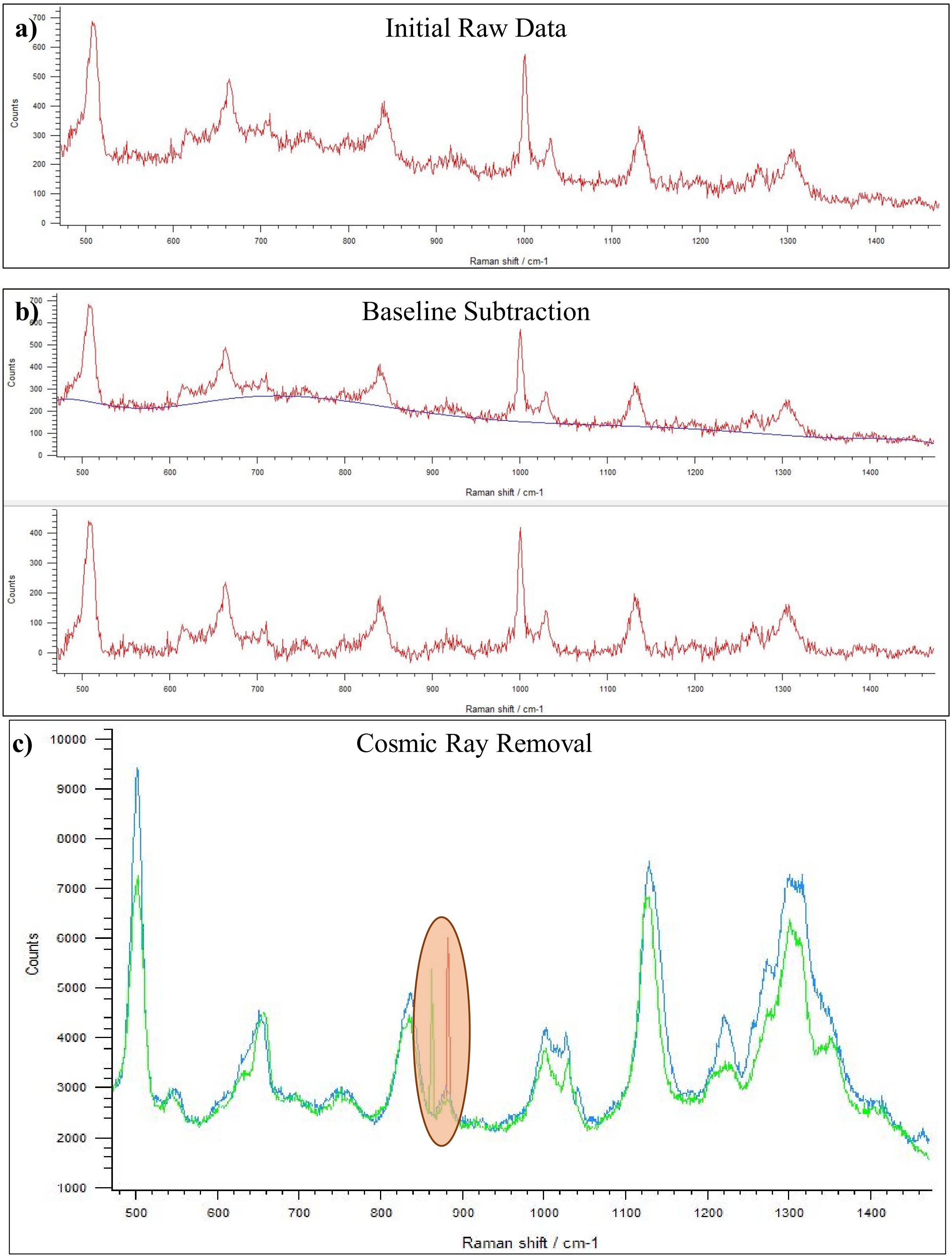
a)
Initial Raw Data
Counts
0 100 200 300 400 500 600 700
500 600 700 800 900 1000 1100 1200 1300 1400
Raman shift / cm-1
b)
Baseline Subtraction
Counts
0 100 200 300 400 500 600 700
500 600 700 800 900 1000 1100 1200 1300 1400
Raman shift / cm-1
Counts
0 100 200 300 400
500 600 700 800 900 1000 1100 1200 1300 1400
Raman shift / cm-1
c)
Cosmic Ray Removal
Counts
1000 2000 3000 4000 5000 6000 7000 9000 10000
500 600 700 800 900 1000 1100 1200 1300 1400
Raman shift / cm-1

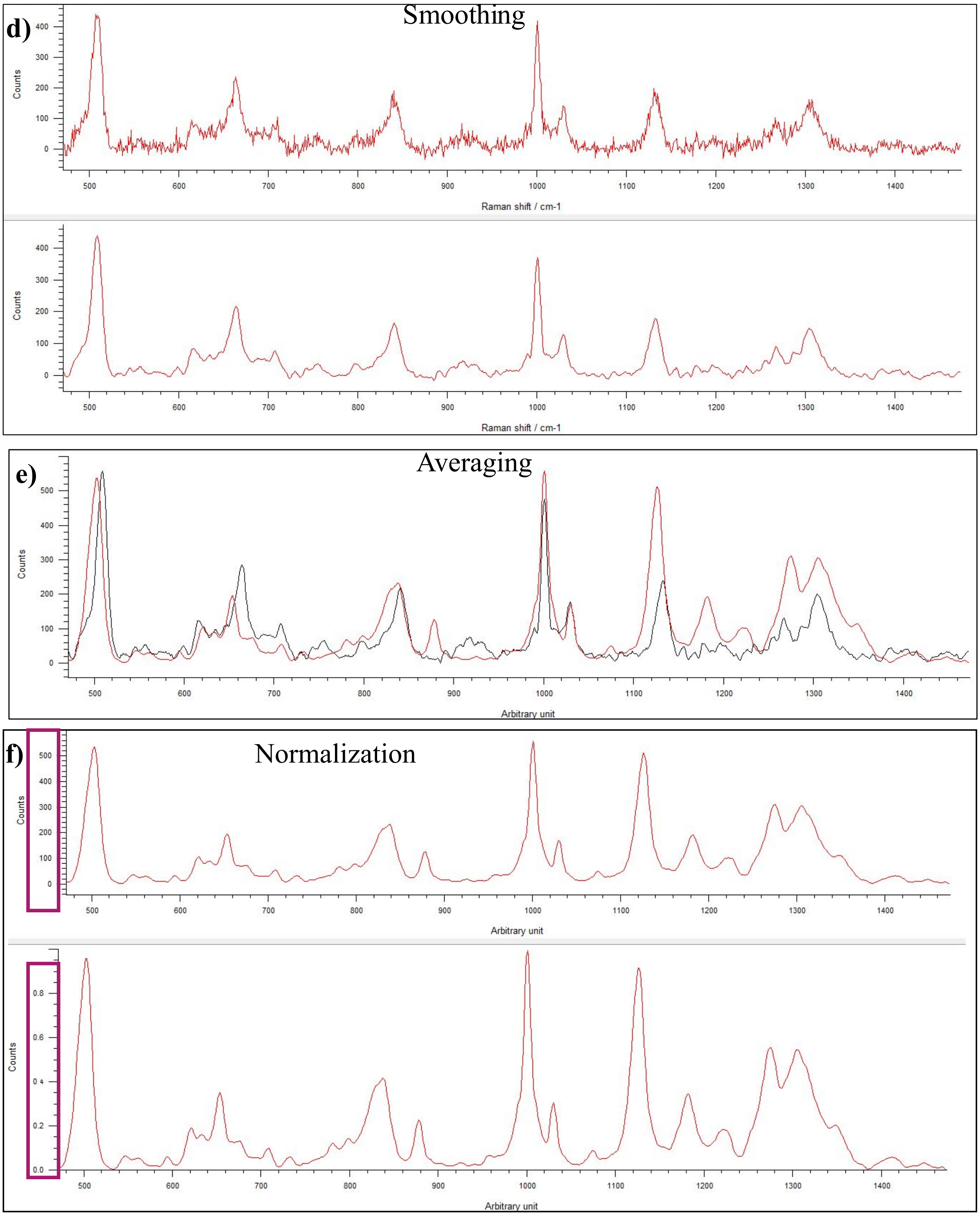

Figure S1. Data analysis steps for SERS measurements for an example spectrum from living cells. a) Raw data, b) Baseline subtraction from the raw data, c) Removal of cosmic ray (removed ray is shown in orange circle), d) Smoothing, e) Averaging approximately 50 spectra from one living single cell and f) Normalization.

Table S1. Tentative SERS Peak Assignments [1-5]

cm^{-1}	Assignment
501	*v*(S-S) gauche-gauche-gauche[3]
548	Cholesterol[1]
573	Cytosine, guanine[1]
596	Phosphatidylinositol[1]
623	C-C twist aromatic ring[1,2]
636	C-S stretching & C-C twisting (Proteins) [3]
653	C-C twisting (Tyrosine)[4]
678	Ring breathing (DNA bases)[2,3]
709	v(C-S) trans[1]
755	Symmetric breathing (Tryptophan) [1,2,3]
800	Phosphate ion interactions[1,2]
838	Ring breathing (Tyrosine) [3]
882	Protein[1,2]
898	Adenine[5]
914	Ribose[1]
1002	Phenylalanine[3]
1012	Ring breathing (Tryptophan) [4]
1030	Phenylalanine, C-N stretching (Proteins)[1,2]
1130	Phospholipid structural changes (trans versus gauche isomerism) [1,3]
1155	C-C (and C-N) stretching (Proteins) [3]
1180	Tyrosine, Phenylalanine[3]
1199	Ring breathing (Tryptophan) [4]
1218	Protein[4]
1272	Protein (α-helix) [3]
1317	Amide III (α-helix) [2]
1352	Protein (β-sheet)[3]

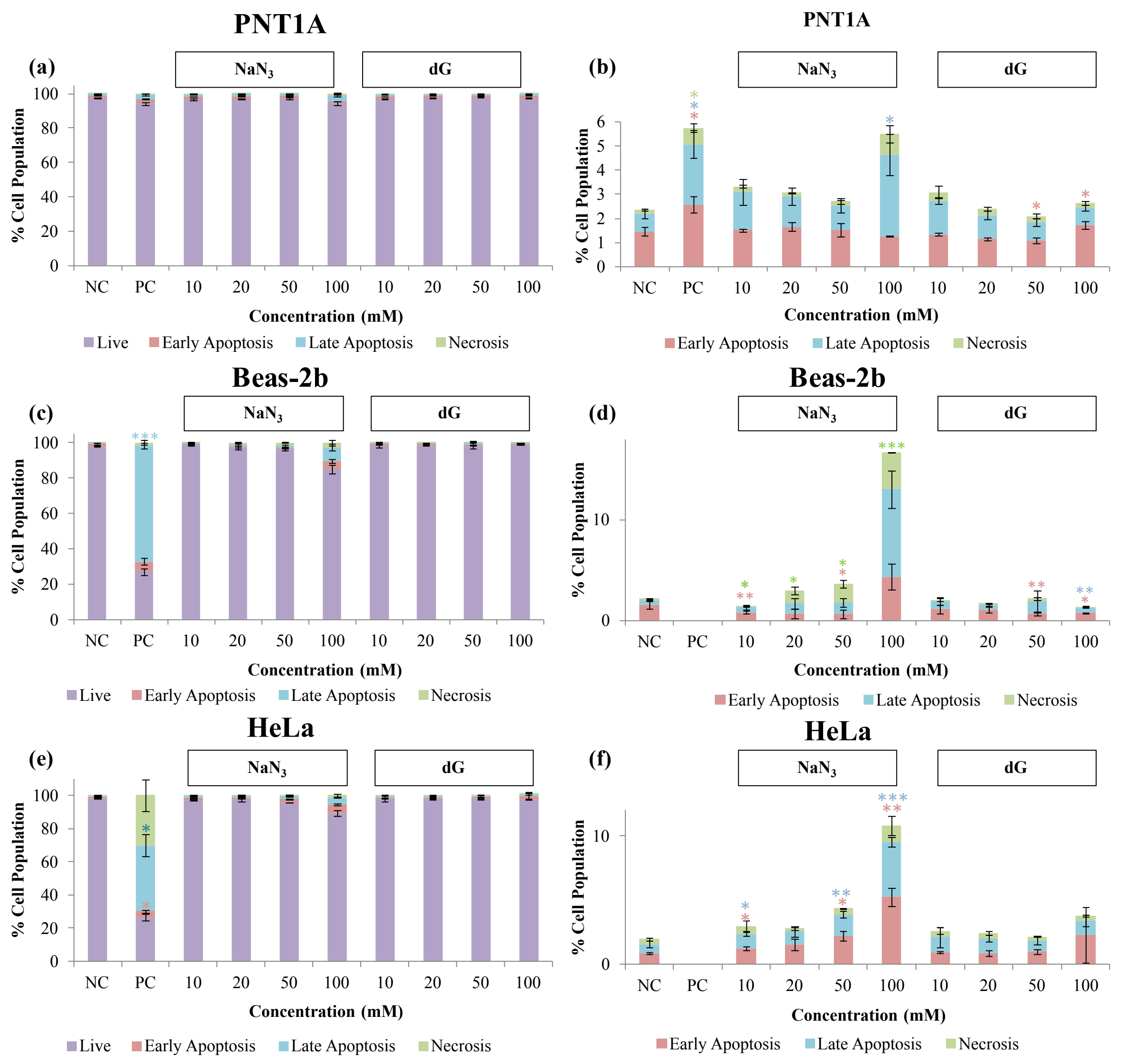

Figure S2. Apoptosis-Necrosis assay result of (a), (b) PNT1A, (c), (d) Beas-2b, and (d), (e) HeLa cells treated with increasing concentrations of NaN_3 and dG. (b), (d) and (e) shows apoptosis and necrosis population in the absence of live population. Statistically significant changes were calculated by two-paired Student's t test, and marked with stars, * for early apoptosis, * for late apoptosis and * for necrosis. * for p≤0.05, ** for p≤0.01 and *** for p≤0.001 with the comparison of normalized control group. PC values were removed from the (d) and (f) for demonstration of statistical values. PC: Positive control (10% DMSO was used as positive control), NC: Negative control.

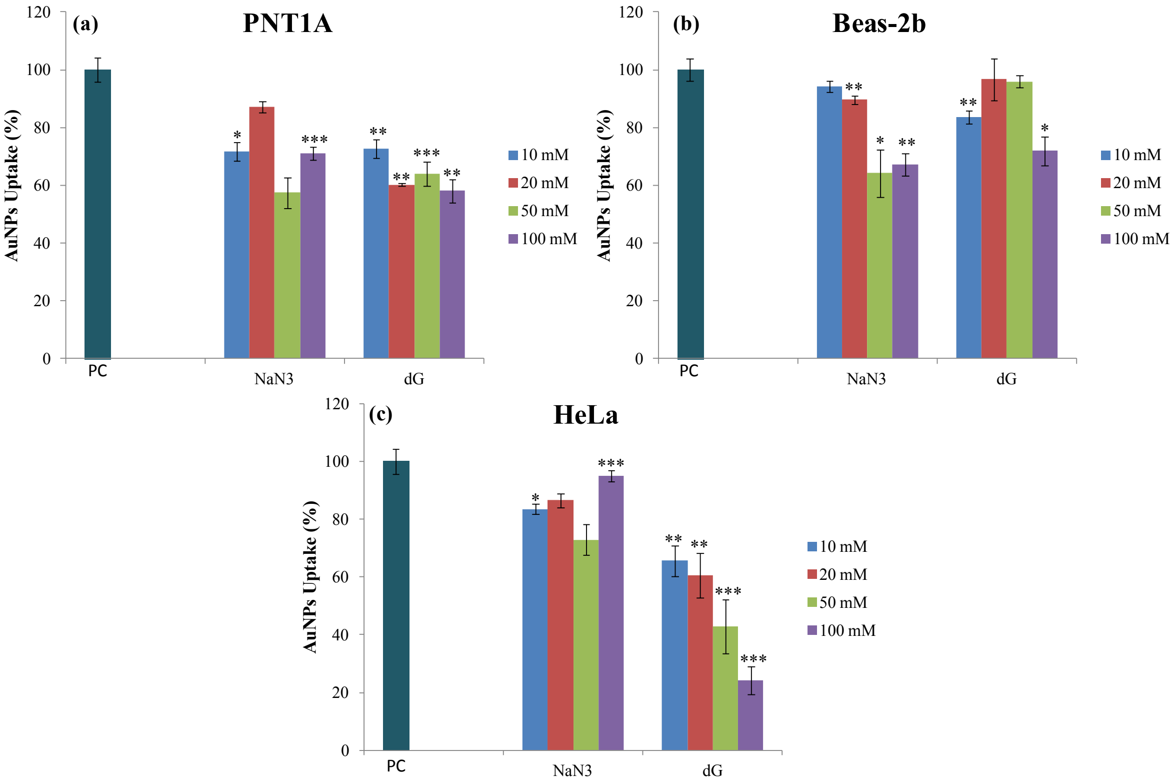

Figure S3. AuNPs internalization data as normalized to SSC shift (%). PC: Positive Control. Statistically significant changes were calculated by two-paired Student's t test, and marked with stars, * for $p \leq 0.05$, ** for $p \leq 0.01$ and *** for $p \leq 0.001$ with the comparison of normalized control group.

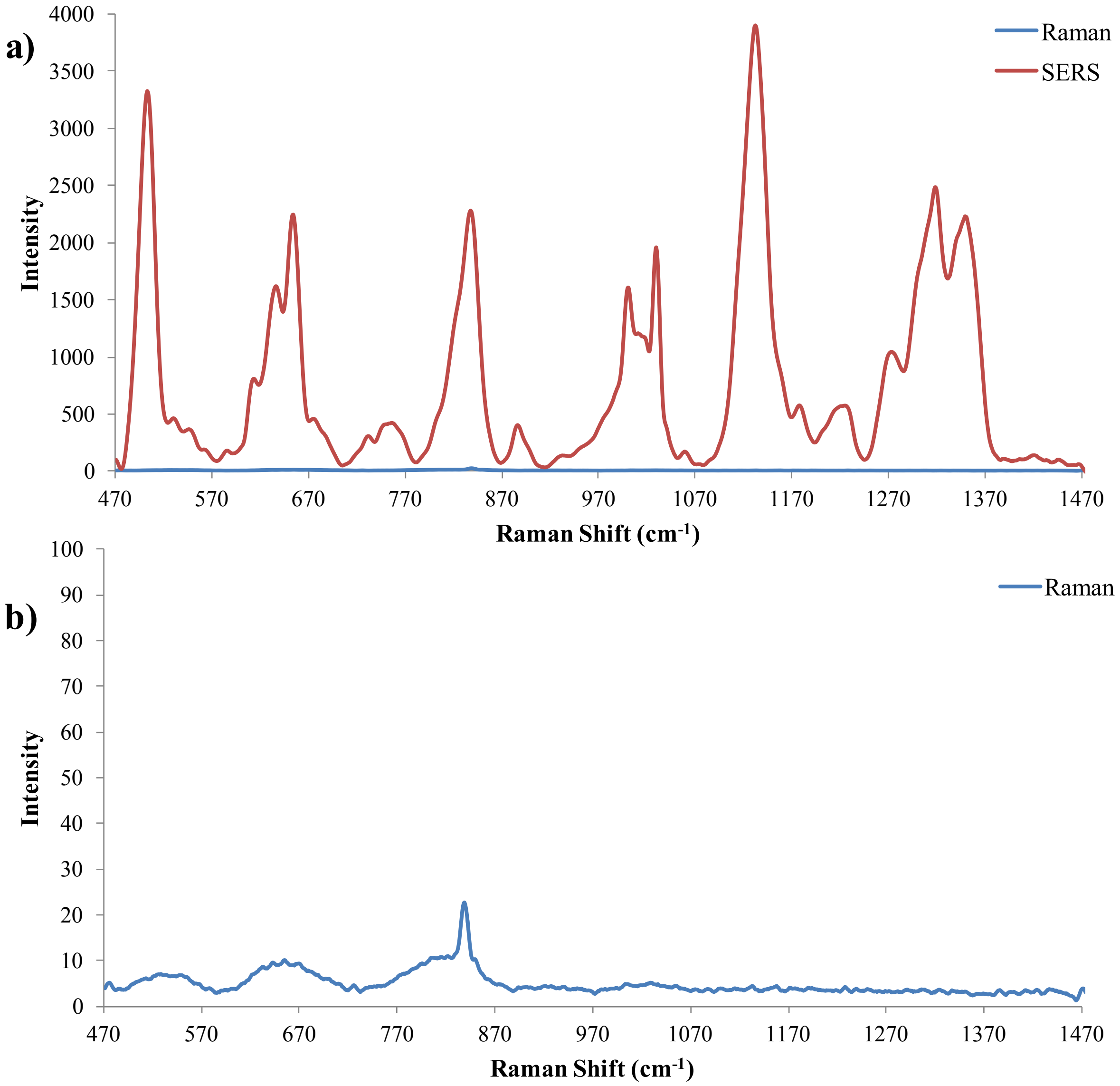

Figure S4. Comparison of SERS (a) and Raman spectra (b) of living cells. Intensity scale adjusted scale to 100 for better visualization of spontaneous Raman spectra of living cells.

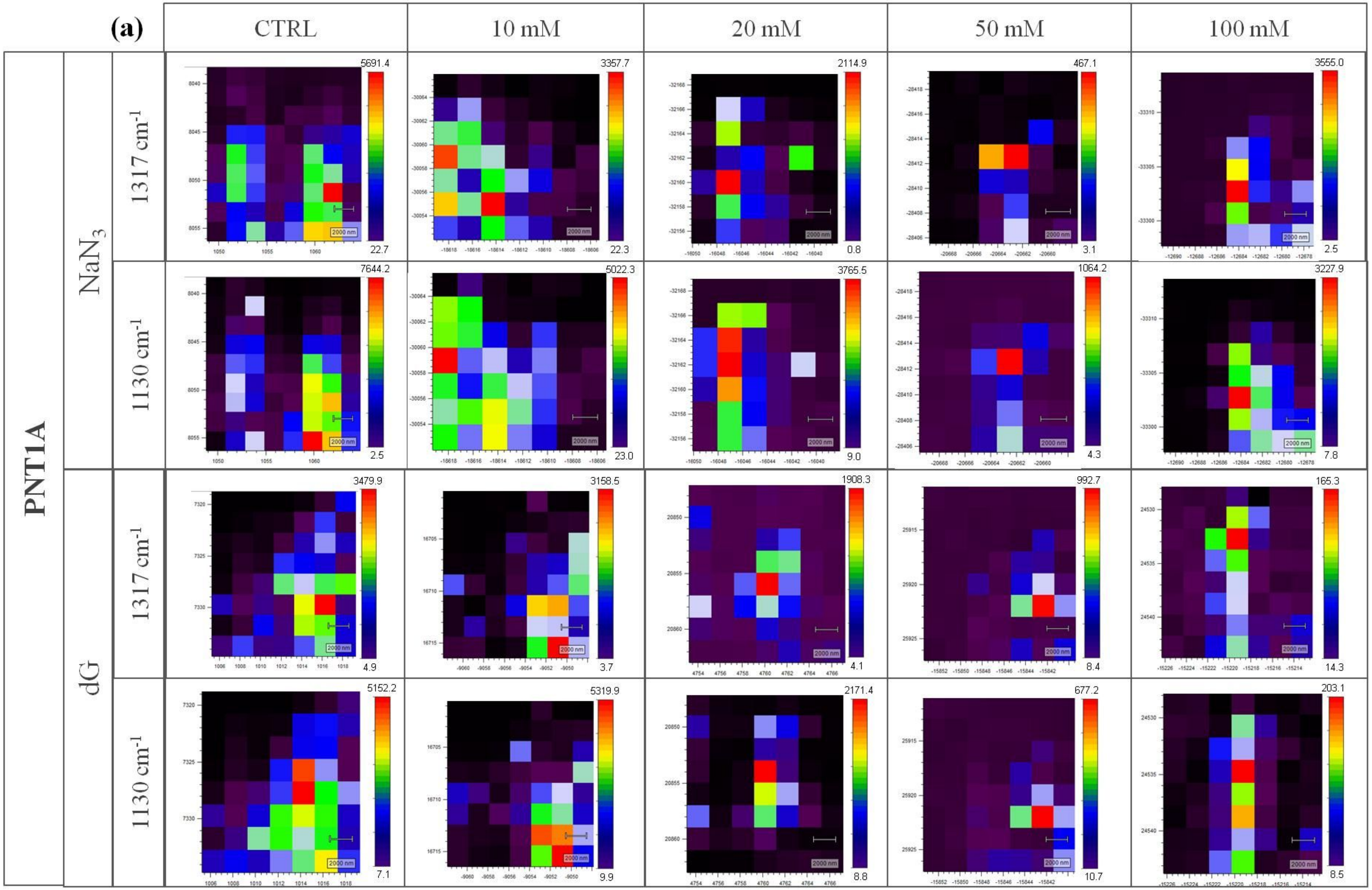
(a)
CTRL
10 mM
20 mM
50 mM
100 mM
PNT1A
NaN3
dG
1317 cm-1
1130 cm-1
5691.4
22.7
3357.7
22.3
2114.9
0.8
467.1
3.1
3555.0
2.5
7644.2
2.5
5022.3
23.0
3765.5
9.0
1064.2
4.3
3227.9
7.8
3479.9
4.9
3158.5
3.7
1908.3
4.1
992.7
8.4
165.3
14.3
5152.2
7.1
5319.9
9.9
2171.4
8.8
677.2
10.7
203.1
8.5
2000 nm

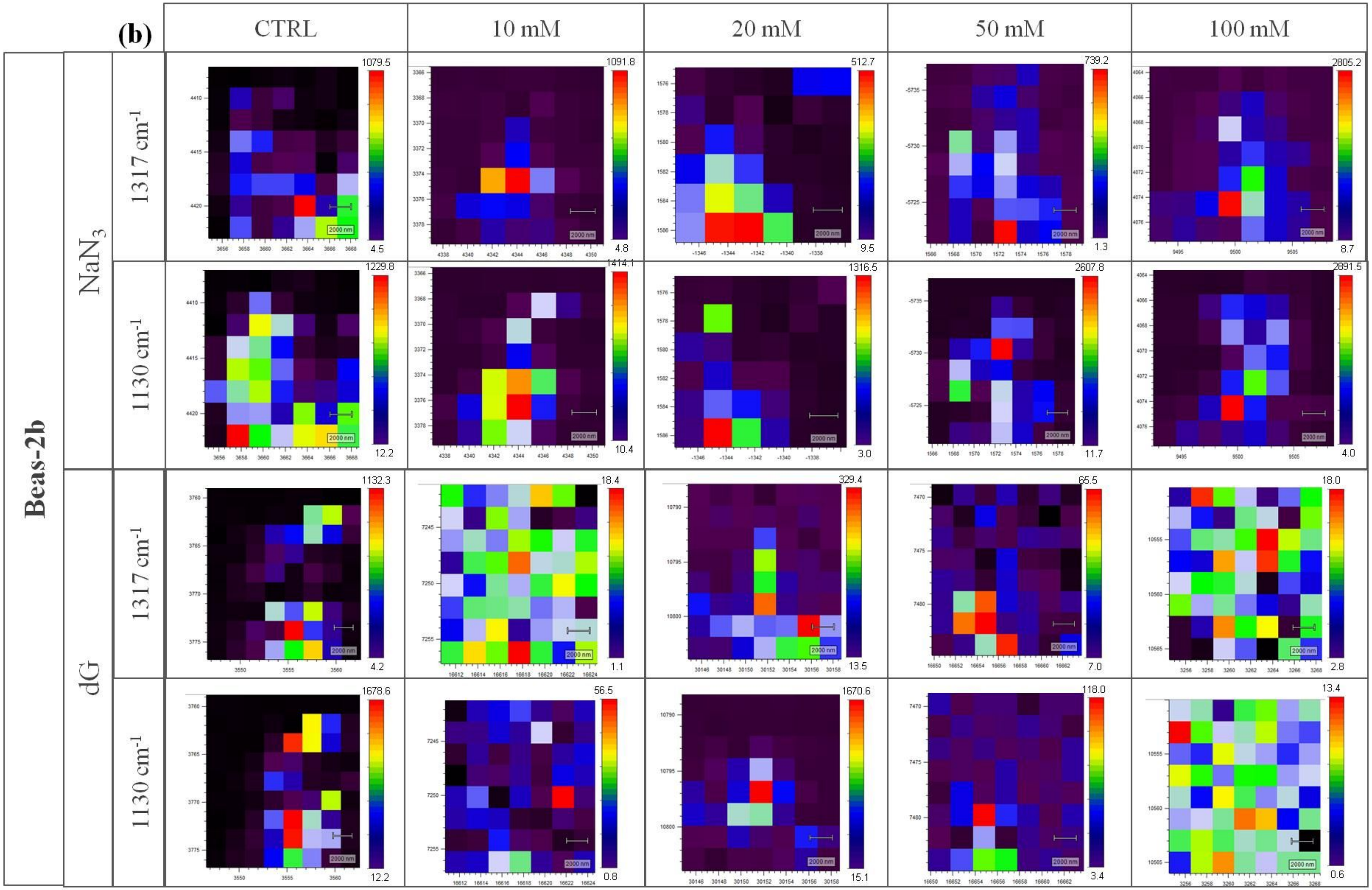
(b)
CTRL
10 mM
20 mM
50 mM
100 mM
Beas-2b
NaN3
dG
1317 cm-1
1130 cm-1
1317 cm-1
1130 cm-1

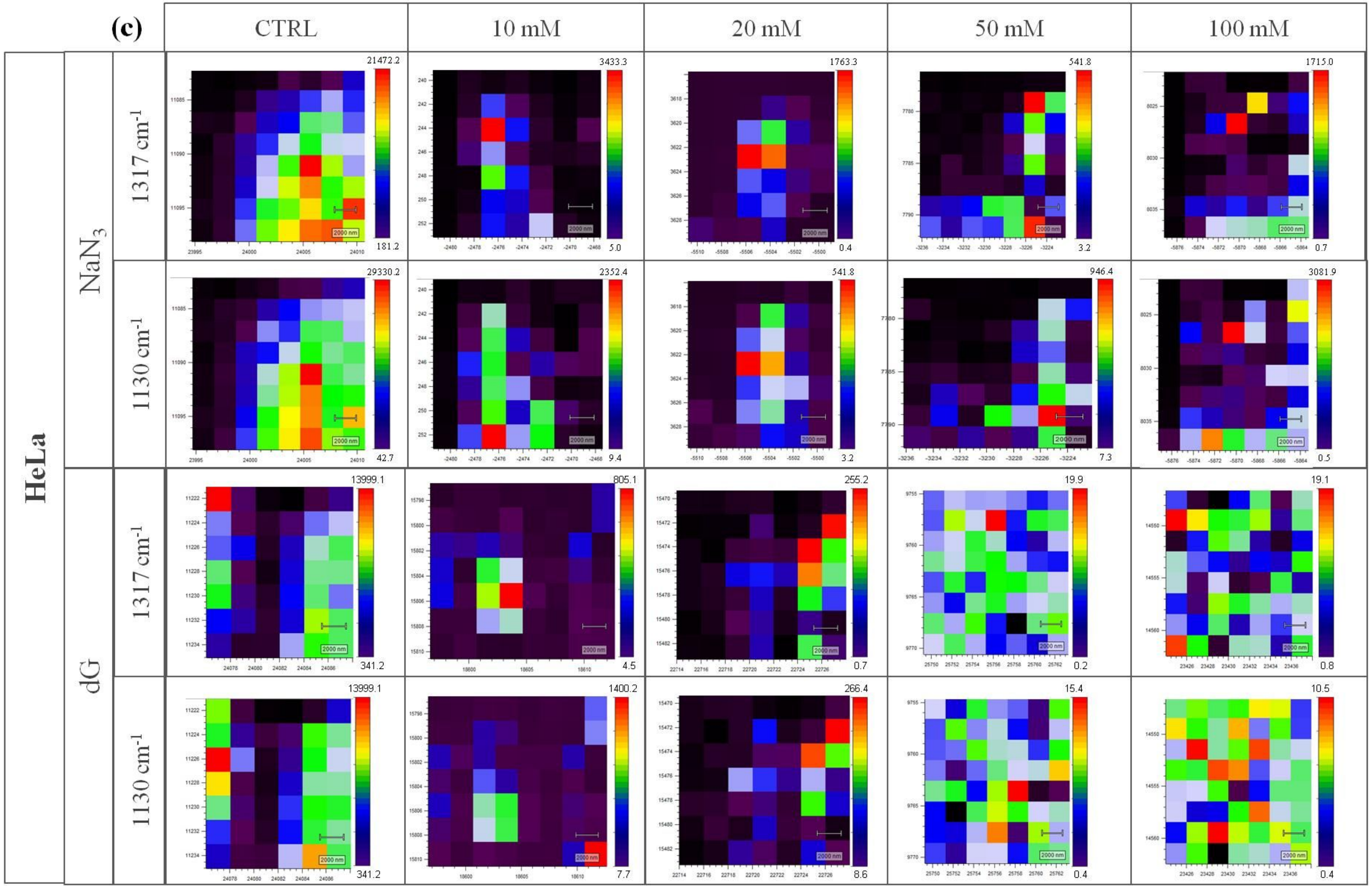

Figure S5. Representative map data of a) PNT1A, b) Beas-2b and c) HeLa cells treated without any inhibitor (CTRL) and 10, 20 50, and 100 mM NaN_3 or dG.

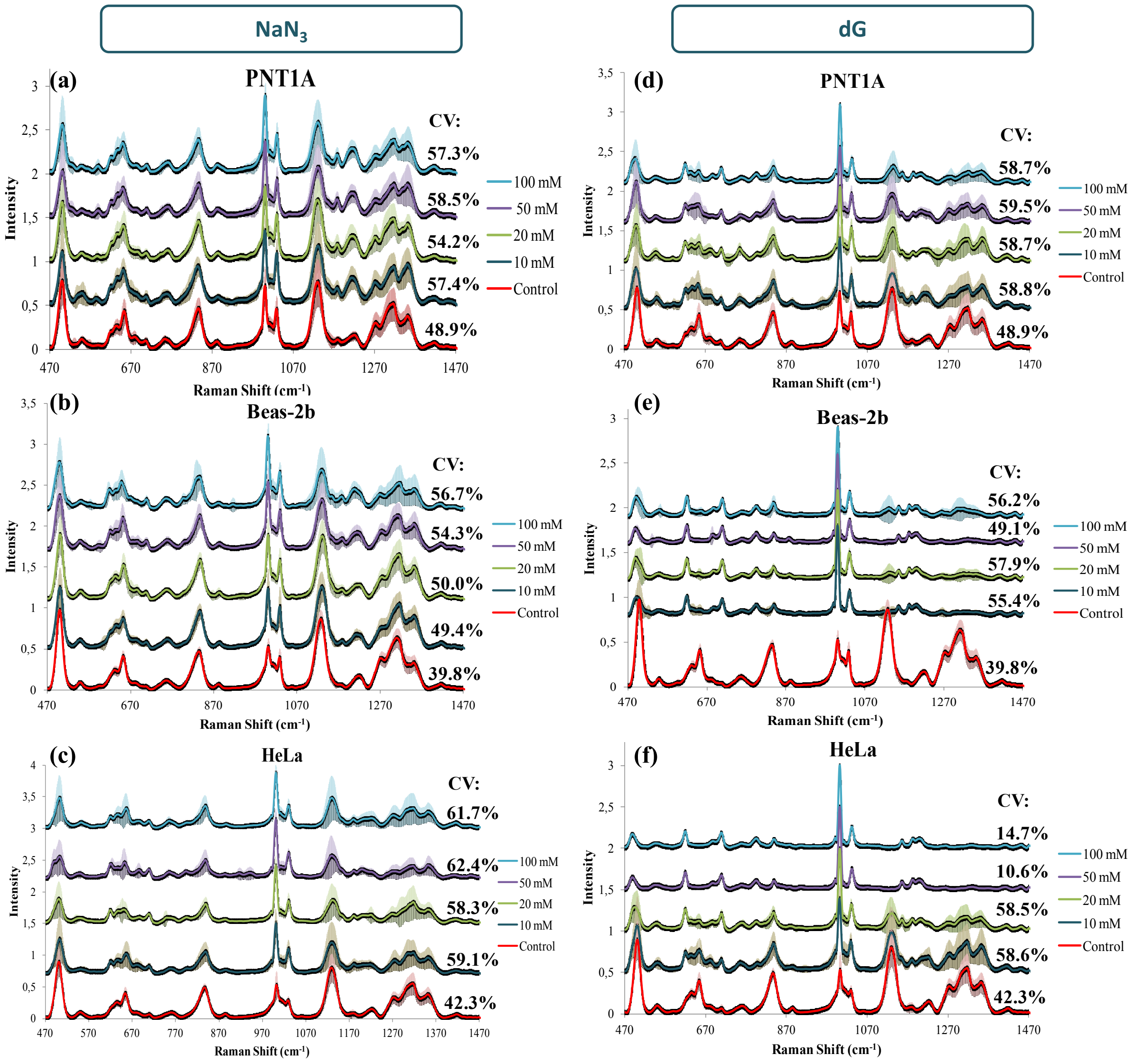

Figure S6. Variations in intracellular SERS spectra along with coefficient of variance (CV) values with exposure to a-c) NaN_3 and d-f) dG of a, d) PNT1A, b, e) Beas-2b, c, f) HeLa cells, respectively.

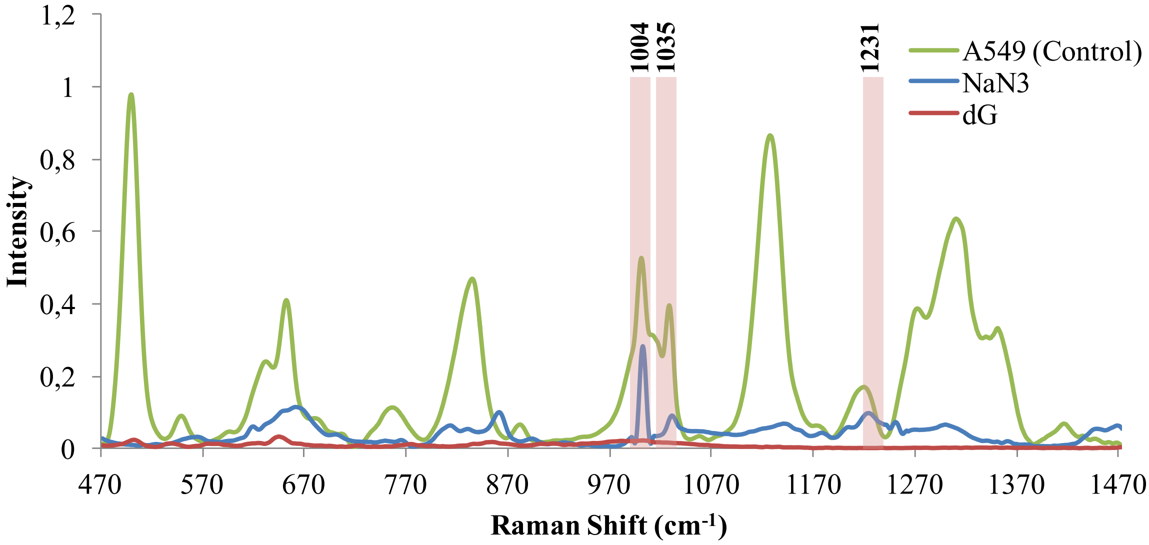

Figure S7. Comparison of SERS Spectra of NaN_3 (100 mM) and dG (100 mM) to SERS spectra of HeLa cells"

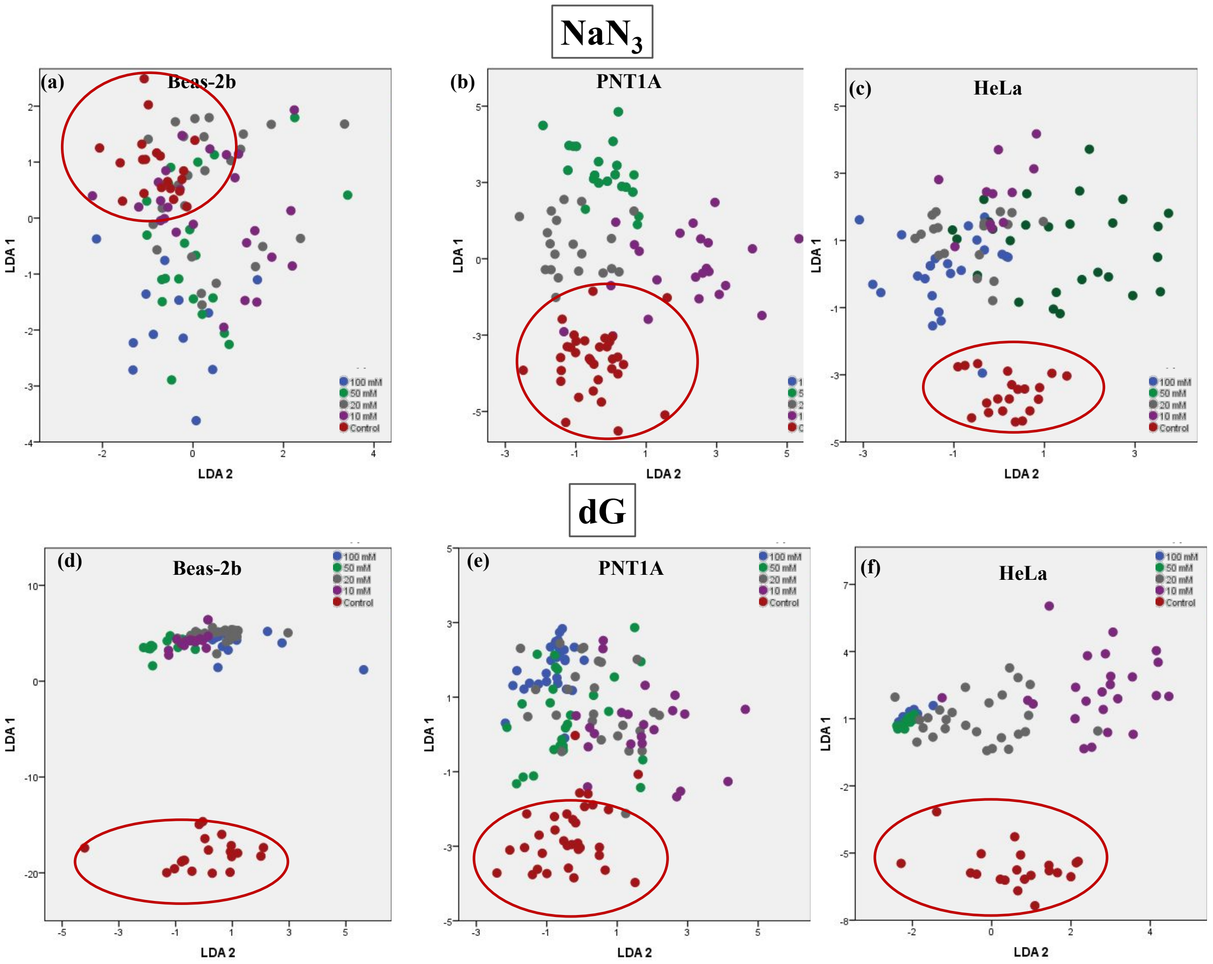

Figure S8. LDA Plots of 10, 20, 50 and 100 mM NaN_3 treated (a) Beas-2b, (b) PNT1A, (c) HeLa cells and dG treated (d) Beas-2b, (e) PNT1A, (f) HeLa cells

REFERENCES

1. Movasaghi, Z.; Rehman, S.; Rehman, I. U. Raman spectroscopy of biological tissues, *Applied Spectroscopy Reviews*. **2007**, 42(5), 493-541. https://doi.org/10.1080/05704920701551530
2. Öztaş, D. Y.; Altunbek, M.; Uzunoglu, D.; Yılmaz, H.; Çetin, D.; Suludere, Z.; Çulha, M. Tracing size and surface chemistry-dependent endosomal uptake of gold nanoparticles using surface-enhanced Raman scattering, *Langmuir,* **2019** 35(11) (2019) 4020-4028. https://doi.org/10.1021/acs.langmuir.8b03988
3. Kuku, G.; Saricam, M.; Akhatova, F.; Danilushkina, A.; Fakhrullin, R.; Culha, M.; Surface-enhanced Raman scattering to evaluate nanomaterial cytotoxicity on living cells, Analytical chemistry. 88(19) (2016) 9813-9820. https://doi.org/10.1021/acs.analchem.6b02917
4. G. Kuku. Development of surface-enhanced Raman scattering-based nanospectroscopic methods for toxicity determination of nanomaterials. Ph.D. Dissertation, Yeditepe University, Istanbul, Turkey, 2017. https://tez.yok.gov.tr/UlusalTezMerkezi/tezDetay.jsp?id=Ewy-awt42-Znw3cFmTos5A&no=Jdx21c6sQW_zKzZtho126Q
5. Shipp, D.W.; Sinjab, F.; Notingher, I. Raman spectroscopy: techniques and applications in the life sciences. *Advances in Optics and Photonics,* **2017**, 9(2), 315-428.
